# Dissolution Behavior of Niobium Compounds under Alkaline Conditions

**DOI:** 10.1021/acsomega.6c05225

**Published:** 2026-07-20

**Authors:** Cássia Ribeiro Souza, Frederico Marques Penha, Sônia Denise Ferreira Rocha

**Affiliations:** † Department of Mining Engineering, School of Engineering, Universidade Federal de Minas Gerais (UFMG), Avenida Presidente Antônio Carlos, 6627 Belo Horizonte, Minas Gerais, Brazil; ‡ Department of Chemical Engineering, KTH Royal Institute of Technology (KTH) CBH School, Teknikrigen 42, 11428 Stockholm, Sweden

## Abstract

This study investigated the dissolution of niobic acid (Nb_2_O_5_·*n*H_2_O) and potassium niobate (mix of phases K_4_Nb_6_O_17_ and KNbO_3_) using the polythermal method in different concentrations of a KOH solution (0–2 mol/L). The software OLI Studio Stream Analyzer and PHREEQC were used for the thermodynamic modeling. The equilibrium time of niobic acid dissolution in an alkaline medium was investigated. Aqueous niobium concentration was measured by ICP-OES and UV–vis spectroscopy methods. The measured dissolved Nb concentrations at 30 °C show deviations from predicted values, indicating the lack of niobium solubility data. The experimental results from the polythermal method evidenced that the dissolution of niobic acid increases with temperature and decreases with KOH concentration. Nevertheless, potassium niobate exhibited a complex behavior, with different phases dissolving preferentially under the studied conditions (0–1 mol/L). Our findings contribute to the understanding of niobium chemistry in alkaline medium, crucial for the development of hydrometallurgical processing of niobium primary sources, which is directly related to the efficiency of leaching processes, crystallization control, and subsequent recovery steps of secondary sources.

## Graphical abstract

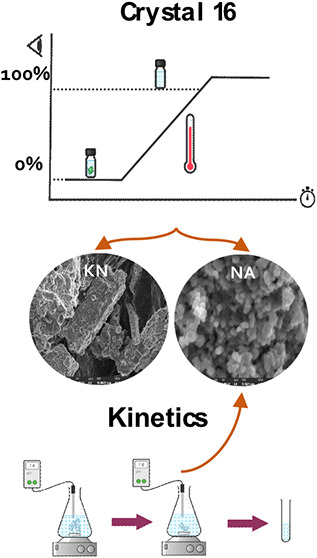

## 1 Introduction

Niobium (Nb) is a transition metal in group V of the periodic table. Most available reports indicate that niobium oxidation states from +I to +V are possible, but according to Filella and May,^1^ niobium oxidation up to +VII is possible. Additionally, only Nb­(V) exists under usual redox conditions in biological and environmental systems. It is a refractory metallic solid of lower density (8.57 g/cm^3^), has high thermal and electrical conductivity,^2^ and is soft and ductile with a very high melting point of 2468 °C^3^ and high resistance to corrosion as a result of surface passivation by oxide film.^4^ Recently, niobium compounds have received attention due to their broad applications in electric batteries, optical films and lenses, semiconductor and microelectronic components, and also as chemical products for catalysis.^5–8^

Potassium niobate has piezoelectric and photorefractive properties that are of interest for the study of ferroelectric phase transition^9^ and can substitute lead zirconate titanate (PZT).^10,11^ There are also considerable applications for potassium niobate in ceramics and glasses.^12–14^ In addition, due to their anisotropic structure, potassium niobate oxides have been studied for their photocatalytic properties, especially K_4_Nb_6_O_17_.^15^ The use of niobium pentoxide as an electronic material has also emerged as a promising material due to its resistance to acids and bases, stability in aqueous medium, and high refractive index.^16^ These properties have motivated its application in energy technology, including lithium-ion battery anodes, photovoltaic devices, photocatalytic systems, sensors, and optical components.^16–21^

Owing to its complexity, the chemistry of niobium has been under investigation;^1^ however, data are still scarce in the literature. For these systems, aqueous speciation diagrams are the most employed tool to predict the effect of pH and total niobium concentration on distinct species formation. Although some studies on niobium solubility are available in the literature (Table 1), data on the solubility of niobium compounds in aqueous media under specific conditions remain scarce, which justifies an effort to access them. Understanding the aqueous chemistry of niobium compounds, particularly their solubility, is essential for advancing research and technological applications for diverse compounds in the field.

**Table 1:** Equilibrium Constants Values for Hydro-Complexes of Niobium at 25 °C^a^

reaction	log *K*		reference
$\mathrm{Nb}{\left(\mathrm{OH}\right)}_{5\left(\mathrm{aq}\right)}+H_{2}O=\mathrm{Nb}(\mathrm{OH}{)}^{6-}+H^{+}$	–6.32	Yajima et al., citated by Fillela and May, 2020	
	–6.76		
$\mathrm{Nb}{\left(\mathrm{OH}\right)}_{5\left(\mathrm{aq}\right)}=\mathrm{Nb}{{\left(\mathrm{OH}\right)}_{4}}^{+}+{\mathrm{OH}}^{-}$	–14.60		Babko et al.
$\mathrm{Nb}{\left(\mathrm{OH}\right)}_{5\left(\mathrm{aq}\right)}={{\mathrm{NbO}}_{3}}^{-}+H^{+}+2H_{2}O$	–7.40		
$\mathrm{Nb}{{\left(\mathrm{OH}\right)}_{5}}_{\left(\mathrm{aq}\right)}+2H_{2}O=\mathrm{Nb}{{\left(\mathrm{OH}\right)}_{7}}^{-2}+2H^{+}$	–14.66		ΔG_f_ data from Peiffert et al.
$7{\mathrm{Nb}}_{6}{O_{19}}^{8-}+2H^{+}=6{\mathrm{Nb}}_{7}{O_{22}}^{9-}+H_{2}O$	3.95		Petrus et al.
$$5{\mathrm{Nb}}_{6}{O_{19}}^{8-}+22H^{+}=3{\mathrm{Nb}}_{10}{O_{28}}^{6-}+11H_{2}O$$			
$4{\mathrm{Nb}}_{6}{O_{19}}^{8-}+17H^{+}=H_{9}{\mathrm{Nb}}_{24}{O_{72}}^{9-}+4H_{2}O$	185.29		
$6\mathrm{Nb}{{\left(\mathrm{OH}\right)}_{4}}^{+}={\mathrm{Nb}}_{6}O{19_{8}}^{-}+14H^{+}+5H_{2}O\left(l\right)$	–70.54		Noseck et al.
$6\mathrm{Nb}{\left(\mathrm{OH}\right)}_{4}+={\mathrm{HNb}}_{6}{O_{19}}^{7-}+13H^{+}+5H_{2}O\left(l\right)$	–56.54		
$6\mathrm{Nb}{{\left(\mathrm{OH}\right)}_{4}}^{+}=H_{2}{\mathrm{Nb}}_{6}{O_{19}}^{6-}+12H^{+}+5H_{2}O\left(l\right)$	–43.54		
$6\mathrm{Nb}{{\left(\mathrm{OH}\right)}_{4}}^{+}=H_{3}{\mathrm{Nb}}_{6}{O_{19}}^{5-}+11H^{+}+5H_{2}O\left(l\right)$	–31.94		
${\mathrm{Nb}}_{6}{O_{19}}^{8-}+H^{+}={\mathrm{HNb}}_{6}{O_{19}}^{7-}$	16.11		Etxebarria et al. and Petrus et al.
	11.87		
${\mathrm{Nb}}_{6}{O_{19}}^{8-}+2H^{+}=H_{2}{\mathrm{Nb}}_{6}{O_{19}}^{6-}$	27.97		
	23.21		
${\mathrm{Nb}}_{6}{O_{19}}^{8-}+3H^{+}=H_{3}{\mathrm{Nb}}_{6}{O_{19}}^{5-}$	39.91		
	32.80		

a [1] Collected the thermodynamic data available in a variety of systems containing different anions (F^–^, Cl^–^, Na^+^, NO_3‑_, C_2_O_4_^–^, C_3_H_2_O_4_^2–^, and C_4_H_4_O_4_^2–^).

In pure aqueous media, Nb­(V) is stable in the water stability field,^22–24^ while Nb­(III) is unstable and rapidly oxidizes to Nb­(V).^25^ Nb­(V) complexes can exist in alkaline and acidic media with a tendency to hydrolyze due to their low stability in aqueous solutions and strong oxygen bonds.^24,26^ Additionally, these complexes produce insoluble oxides with amphoteric behavior under both acidic and basic conditions. These oxides produce anionic species above pH 4 and cationic species below pH 4.^7^

Mononuclear species of Nb­(V) can exist in aqueous media as Nb­(OH)_3_^2+^, Nb­(OH)_4_^+^, Nb­(OH)_7_^2–^, Nb­(OH)_5_, and Nb­(OH)_6_^–^. Jo et al.^22^ described that due to the increased solubility of Nb­(V) in a pH higher than 12.5, elevated Nb concentration enables the formation of hydrolyzed species of Nb­(V), in addition to Nb­(OH)_7_^2–^ and in pH values higher than 14.5, the formation of NbO_2_(OH)_4_^3–^ species is predicted. It must be noted that most mononuclear species of Nb­(V) are theoretical and have not been directly observed spectroscopically; only polynuclear species have been experimentally confirmed.^27^

In an alkaline medium, niobium knowingly forms Lindqvist-type ions H_*x*_M_6_O_19_^(*x*–8)^ (M = metal), and the formation of the M_6_O_19_ cluster has been reported.^24,27^ This structure (H_*x*_Nb_6_O_19_^(*x*–8)^) is the dominant species for Nb­(V) in an aqueous medium in pH ≥ 8, related to hexaniobate salt formation.^24,28–31^ The hexaniobate ions are also precursors for several polyoxoniobates, including Nb_10_O_28_^6–^, Nb_20_O_54_^8–^, and Nb_24_O_72_H_91_^5–^.^32^

Petrus et al.^27^ predicted, in the speciation diagram, the formation of new species Nb_10_O_28_^6–^, Nb_7_O_22_^9–^, and H_9_Nb_24_O_72_^9–^, with Nb_10_O_28_^6–^ species being the predominant from acidic to neutral pH values. H_9_Nb_24_O_72_^9–^ is also formed at similar pH values but in higher niobium concentrations. Therefore, Nb_7_O_22_^9–^ is probably a transient intermediate and rapidly reacts to form the aforementioned species. Additionally, Jehng and Wachs^26^ speculate that hexaniobate species begin to degrade into tetrameric (Nb_4_O_16_^12–^) and monomeric (NbO_2_(OH)_4_^3–^) species in alkaline and dilute solutions.

According to Włódarkiewicz et al.,^33^ pH strongly governs the solubility and niobate salts may dissolve incongruently, releasing primarily alkali cations, and the dissolution typically follows the order [K^+^] > [Na^+^] ≫ [Nb^5+^].

The hexaniobate ion is an excellent precursor for the synthesis of heteropolyoxoniobates. When associated with elements such as K, Na, Rb, Cs, and Li (reaction rate: Cs > Rb > K > Na > Li), these can be introduced in the Lindqvist structure and produce new species and compounds, e.g., the potassium ion, for example, can form K_4_(Nb_6_O_19_)^4–^, K_8_(Nb_6_O_19_), and K_10_(Nb_6_O_19_)^2+^.^28,34^ The chemical reactions of niobium complexes in aqueous media and their equilibrium constants are presented in Table 1.

In an acidic medium, niobic acid, a hydrated niobium oxide (Nb_2_O_5_·nH_2_O), is formed, with low solubility in water. After calcination, anhydrous niobium pentoxide is obtained. Niobic acid is more reactive than its equivalents and can be produced by a hydrometallurgical route,^29^ either by NbCl_5_(aq) neutralization or by precipitation through the acidification of alkaline Nb solutions. Regarding the solubility of niobium compounds, most available literature refers to niobium pentoxide and niobic acid, as compiled in Table 2. It is important to highlight that the high variability in solubility observed in the table is due to the different experimental conditions of each study.

**Table 2:** Solubility Studies for Niobium Compounds

		solubility		
compound	compound origin	Nb (mol/kg)	condition	refs
Nb_2_O_5_	Precipitation with KNO_3_	1.4 × 10^–5^	*I* = 1–6 mol/L KNbO_3_, 19 °C; pH = 0–7	Babko et al.
Nb_2_O_5_	Precipitation (reagents not reported)	1 × 10^–8^	*I* = 0.1 mol/L NaCl; 25 °C pH = 2–8	Yajima et al. and Yajima – as cited by Lothenbach et al.
Nb_2_O_5_	Recrystallization	6 × 10^–10^	*I* = 0.1–6 mol/kg NaClO_4_; 10–70 °C, pH = 1–9 monoclinic phase	Peiffert et al.
Nb_2_O_5_	Not reported	∼10^–4^ to 10^–7^	0.1 – 1 mol/kg NaOH and Na_2_CO_3_, 550 °C	Kotova
Nb_2_O_5_	Not reported	10^–4^ to 10^–3.5^	10^–2^ mol/kg HF and >1 mol/kg HF at 550 °C, 1000 bar, Co-CoO oxidizing medium	Kotova
		10^–1.5^ to 10^–0.5^		
Nb_2_O_5_	Not reported	10^–5^ to 10^–4^	0.1 and 1 mol/kg HCl and KCl, 300 and 500 °C, 100 MPa Co-CoO oxidizing medium	Kotova
Nb_2_O_5_	Not reported	∼10^–7^ to 10^–8^	*I* = 10^–5^ a 10^–4^ mol/L HF, 150, 200, and 250 °C; pH < 3	Timofeev et al.
Nb_2_O_5_·nH_2_O	Hydrolysis with NbCl_5_	5.4 × 10^–6^	*I* = 0.12 mol/L NaCl/NaOH/Na_2_CO_3_; 25 °C; pH = 6–12	Deblonde et al.
Na_7_HNb_6_O_19_·15H_2_O	Hydrothermal Nb_2_O_5_ and KOH	∼10^–3^ (*K* = 2.275 × 10^–12^)	*I* = 0.09–0.18 NaCl/KCl; 25 °C; pH = 11–12	

As can be seen in Table 2, niobium oxide has low solubility in an aqueous medium under both basic and acidic pH conditions, with the exception of hydrofluoric acid solution, which increases the solubility of niobium due to the lower tendency to form polymeric species.^39^

Babko et al.^40^ investigated the solubility of freshly precipitated niobium hydroxide (probably an amorphous phase) in the presence of HNO_3_. This study was used by Etxebarria et al.^41^ to estimate the solubility product of niobium pentoxide. On the other hand, Lothenbach et al.^42^ used the data of Yajima et al. and Yajima on the Nb_2_O_5_ solubility in a NaCl solution to calculate solubility products from this study (publications of Yajima et al. and Yajima not available)

Peiffert et al.^7^ studied the effect of temperature, ionic strength, and pH on the solubility of the monoclinic phase of Nb_2_O_5_ obtained at moderate temperatures (∼600–900 °C). The results show an increase in solubility with an increase in temperature and ionic strength, and the presence of polyniobates was reported at higher pH values.

Kotova^36,37,43^ studied the solubility of niobium oxide in the presence of different electrolytes, such as NaOH, Na_2_CO_3_, KCl, HCl, and HF. Co-CoO buffer with low oxygen fugitiveness was used due to the negative influence of an increase in oxygen fugitiveness on the solubility.^43^ The solubility in the presence of NaOH and Na_2_CO_3_ was low, on the order of 10^–4^ to 10^–7^ mol/L, and the effect of niobium chloride complexes on niobium solubility was insignificant. However, the solubility of Nb_2_O_5_ increases strongly with increasing concentrations of fluoride solutions.

Timofeev et al.^38^ also investigated the solubility of Nb_2_O_5_ in HF solutions at lower pH values by adding HClO_4_ at 150, 200, and 205 °C. They also noticed that a decrease in the pH increased the solubility of niobium oxide at low HF concentrations. However, for niobates, there is a lack of solubility data in the literature. To the best of our knowledge, only Deblonde et al.^29^ studied the solubility of a sodium niobate phase in the NaCl/KCl medium at 25 °C. This study shows the effect of the common ion on solubility, where an increase in Na^+^ causes a decrease in the solubility of sodium niobate. In a Na^+^ medium, the dissolution behavior is mainly governed by the common-ion effect, with no significant ion-pair formation between Na^+^ and the hexaniobates species. In contrast, the K^+^ ion interacts more strongly with the hexaniobates species through ion-pair formation, stabilizing dissolved niobium species and increasing the apparent solubility. Additionally, the solubility of niobic acid increased with the increase in pH, and a major role of hexaniobate ions in the solubility of niobic acid in an aqueous medium was also pointed out.

In this context, this work focused on investigating the dissolution, as an approximation for the solubility of niobic acid (Nb_2_O_5_·*n*H_2_O) and potassium niobate (mixed phases: K_4_Nb_6_O_17_ and KNbO_3_) at different temperatures and concentrations of KOH solutions. Additionally, dissolution experiments for both potassium niobate and niobic acid in alkaline media (0–2 mol/L KOH) were performed using Crystal 16 equipment and compared with solubility results from thermodynamic modeling conducted for the system Nb–K–H_2_O in OLI Studio Stream Analyzer and PHREEQC.

Due to the experimental approach employed, the structural characteristics of the final phases in equilibrium were not determined. Therefore, experimental measurements represent the dissolution behavior of niobium compounds. However, these results may be interpreted as a close approximation of niobium solubility under the conditions studied. Despite this limitation, the results represent a relevant contribution to the understanding of the Nb–K–H_2_O system, especially considering the scarcity of experimental solubility data for niobium compounds in alkaline media.

## 2 Methods

### 2.1 Reagents

Potassium niobate (mixed-phase) was synthesized as described by Souza et al.^8^ via cooling crystallization (100–30 °C) from a liquor obtained from Fe–Nb alloy fines leached with KOH. The mixed-phase K_4_Nb_6_O_17_ and KNbO_3_ were confirmed by XRD. Niobic acid HY-340 Standard was supplied by CBMM (Brazilian Company of Metallurgy and Mining, from Portuguese: *‘Companhia Brasileira de Metallurgia e Mineração’*). Analytical grade NaOH(s) (Êxodo Científica, CAS: 1310–73–2) and KOH(s) (Sigma-Aldrich, CAS: 1310–58–3) were used without further purification. Solutions were prepared with deionized and/or distilled water.

### 2.2 Thermodynamic Modeling

The solution description parameters, such as composition data, saturation index, species distribution in solution, and possible solid phases formed in multicomponent systems, were obtained by thermodynamic modeling with OLI Studio Stream Analyzer (version 11) and PHREEQC (version 3.4.12927).^44^ The thermodynamic framework AQ (aqueous model) and MSE (mixed solvent electrolyte) were used in OLI. The AQ framework includes both stable phases, KNbO_3_ and Nb_2_O_5_, and is based on the Bromley–Zemaitis activity model for electrolytes dissolved in water. In contrast, the MSE framework contains only Nb_2_O_5_ and utilizes the MSE activity model, which uses (i) the extended Debye–Hückel term that accounts for long-range interactions and (ii) an UNIQUAC term that accounts for short-range interactions and a middle-range term that includes the ionic interactions. In PHREEQC, the SIT (specific ion interaction theory) database (sit.dat) was used as the aqueous model. The ionic strength of the system was verified to be within the allowed limit (∼3 mol/kg), confirming the validity of the model used.

The speciation diagram of hexaniobates species was made based on the calculations^41^ using the equilibrium constants of the hydrolysis products of Nb­(V) at 25 °C, as a function of pH (Table 1), according to eqs 1–9

1 $${\left[C\right]}_{T}=\left[{\mathrm{Nb}}_{6}O_{19}^{8-}\right]+\left[{\mathrm{HNb}}_{6}O_{19}^{7-}\right]+\left[{H_{2}\mathrm{Nb}}_{6}O_{19}^{6-}\right]+\left[{H_{3}\mathrm{Nb}}_{6}O_{19}^{5-}\right]$$

2 $$\left[{\mathrm{Nb}}_{6}O_{19}^{8-}\right]=\frac{{\left[C\right]}_{T}}{1+\left(K_{1}\cdot \left[H^{+}\right]\right)+\left(K_{2}\cdot \left[{2.H}^{+}\right]\right)+\left(K_{3}\cdot \left[3.H^{+}\right]\right)}$$

3 $$\left[{\mathrm{HNb}}_{6}O_{19}^{7-}\right]=\left[{\mathrm{Nb}}_{6}O_{19}^{8-}\right]\cdot K_{1}\cdot {[H}^{+}]$$

4 $$\left[{H_{2}\mathrm{Nb}}_{6}O_{19}^{6-}\right]=\left[{\mathrm{Nb}}_{6}O_{19}^{8-}\right]\cdot K_{2}\cdot {{[H}^{+}]}^{2}$$

5 $$\left[{H_{3}\mathrm{Nb}}_{6}O_{19}^{5-}\right]=\left[{\mathrm{Nb}}_{6}O_{19}^{8-}\right]\cdot K_{3}\cdot {{[H}^{+}]}^{3}$$

6 $$\alpha_{1}=\frac{\left[{\mathrm{Nb}}_{6}O_{19}^{8-}\right]}{{\left[C\right]}_{T}}$$

7 $$\alpha_{2}=\frac{\left[{\mathrm{HNb}}_{6}O_{19}^{7-}\right]}{{\left[C\right]}_{T}}$$

8 $$\alpha_{3}=\frac{\left[{H_{2}\mathrm{Nb}}_{6}O_{19}^{6-}\right]}{{\left[C\right]}_{T}}$$

9 $$\alpha_{4}=\frac{\left[{H_{3}\mathrm{Nb}}_{6}O_{19}^{5-}\right]}{{\left[C\right]}_{T}}$$

where *C*[*T*] is the total concentration, *K*_1_, *K*_2_, and *K*_3_ are the equilibrium constants and α_1_, α_2_, α_3_, and α_4_ are the concentration fractions.

The dissolution reactions of the niobium compounds studied in this work can be represented by the following reactions (eqs 10–12)­

$${\mathrm{Nb}}_{2}O_{5}.nH_{2}O_{\left(S\right)}+2{\mathrm{OH}}^{-}⇌{{\mathrm{2NbO}}_{3}}^{-}{\mathrm{+ (}n\mathrm{+1)H}}_{2}O$$ 10

11 $$6{\mathrm{KNbO}}_{3}+H_{2}O⇌{\mathrm{Nb}}_{6}{O_{19}}^{8-}{\mathrm{+ 6K}}^{+}{\mathrm{+ 2OH}}^{-}$$

12 $$K_{4}{\mathrm{Nb}}_{6}O_{17\left(s\right)}+4{\mathrm{OH}}^{-}⇌{\mathrm{Nb}}_{6}{O_{19}}^{8-}{\mathrm{+ 4K}}^{+}\mathrm{+ 2HO}$$

### 2.3 Experimental Section

The equilibrium study was performed for the commercial niobic acid HY-340 at different concentrations of the KOH solution. A mass of niobic acid was mixed into 10 mL of KOH solutions with concentrations from 0.10 to 0.50 mol/L in polyethylene vessels (Table 3). The experiments were carried out in a shaker (SOLAB SL-222) at 30 ± 0.5 °C and 200 rpm. The achievement of the equilibrium conditions was assessed by monitoring Nb concentration in solution as a function of time (0–300 h). The samples were filtered using a 0.45 μm syringe filter (MILLIPORE MILLEX-HV PVDF 0.45 μm). The pH measurements were performed conventionally using a Tecnopon mPA 210A pH meter equipped with a Digimed glass electrode (sensitivity up to 96 °C), and the quantification of Nb was performed after the filtration process.

**Table 3:** Conditions of Equilibrium Study of Niobic Acid in 10 mL of Alkaline Medium at 30 ± 0.5 °C and 200 rpm for 312 h

mass of niobic acid (g)	KOH (mol/L)
0.20	0.10
0.20	0.25
0.50	0.50

The Nb concentration in aqueous solution was determined by ICP-OES (PerkinElmer, model Optima 7300DV; detection limits <0.005 mol/L) and UV–vis (BEL-M51 spectrophotometer) methods. Calibration curves for Nb analysis were prepared with standard reference materials. Replicates, as well as blanks and samples of known concentration, were analyzed for quality control. Blank extractions yielded values below the detection limits (DL) of the method (DL < 0.05 mg/L). Lutetium (1 mg/kg) was used as an internal standard to evaluate instrument performance and assay precision, with recoveries within 90–110%. The parameters for the analytical procedure were defined as follows: plasma power 1300 W; plasma flow 15 L/min; auxiliary gas flow 1.0 L/min; pump flow 1.3 mL/min; Burgener MiraMist nebulizer 0.65 L/min; and Scott chamber.

The Nb quantification with UV–vis was performed using a methodology adapted from Deblonde et al.^24^ Each sample was diluted in a 4 M NaOH solution 10 min before the measurement and recorded against the corresponding blank sample using quartz cuvettes. The absorbance (*A*) was measured at 246.5 nm, using an optical path length (*l*) of 1 cm, and the extinction coefficient (ε) was 15900 ± 600 L/mol·cm to Nb_6_O_19_^8–^ and 14300 ± 400 L/mol·cm to HNb_6_O_19_^7–^ species. The Beer–Lambert law was used for the determination of niobium concentration (*c*) according to eq 13.

13 A=ε·c·l

Crystal 16 (Technobis Crystallization Systems, software v. 2.3.2.5300) was used to estimate the dissolution behavior of potassium niobate and niobic acid. The equipment is a multireactor crystallizer consisting of 16 minireactors (4 rows with 4 vials of 1 mL each), in which temperature profiles can be setup and carried out very precisely while measuring the transmission of a laser through the sample (the higher the transmission, the more dissolved the solid). Hence, to determine the dissolution, clear points should be collected based on the transmissivity of each vial for known amounts of dissolved solids at a known temperature. Different masses of solute were analyzed in 1 mL of deionized water and KOH solution (0–2 mol/L). Each cycle of measurements was conducted at a temperature ramp rate from 20 to 70 °C, with a heating rate of 0.1 °C/min and a stirring speed of 700 rpm. A minimum of three cycles (4 rows with 4 vials of 1 mL each, i.e., 48 data points in total) was conducted in each condition described in Table 4 to ensure the reliability of the study. In this study, only the clear points were collected when 100% of transmissivity was achieved.

**Table 4:** Experimental Data for Dissolution of Potassium Niobate and Niobic Acid in Different Concentrations of KOH Solution in a Temperature Ramp Rate from 20 to 70 °C Using Crystal 16

potassium niobate			niobic acid		
	Concentration (mg/mL)			Concentration (mg/mL)	
KOH	MIN	MAX	KOH (mol/L)	MIN	MAX
0	80	300	0.75	5	80
0.10	30	80	1.00	5	60
0.25	10	80	1.50	10	80
0.50	10	80	2.00	10	60
1.00	10	50			

## 3 Results and Discussion

### 3.1 Niobium Speciation

The speciation diagram of niobium in aqueous media was the initial step in understanding the system and is essential for elucidating the formation of niobium complexes. Figure 1a shows the Nb–H_2_O speciation diagram, simulating the hydrolysis of Nb­(V) in a broad pH range at temperatures of 25, 50, and 100 °C. According to the PHREEQC modeling, the aqueous species Nb­(OH)_5_ is dominant at lower pH values. As the pH increases, the species Nb­(OH)_6_^–^ and Nb­(OH)_7_^2–^ become predominant. At 100 °C, the predominance area of these species changes, shifting to lower pH values. However, the hexaniobate species (Nb_6_O_19_^8–^; HNb_6_O_19_^7–^; H_2_Nb_6_O_19_^6–^; H_3_Nb_6_O_19_^5–^) are not available in the PHREEQC database, which may limit its applicability to predictions in highly alkaline conditions; thus, they do not appear in this diagram. Hence, the hexaniobates speciation (Lindqvist ions structure) was calculated considering the equilibrium equations shown in Table 1 at 25 °C^27,41^ (Figure 1b). The PHREEQC-SIT database can be seen in the Supporting Information.

**Figure 1:**
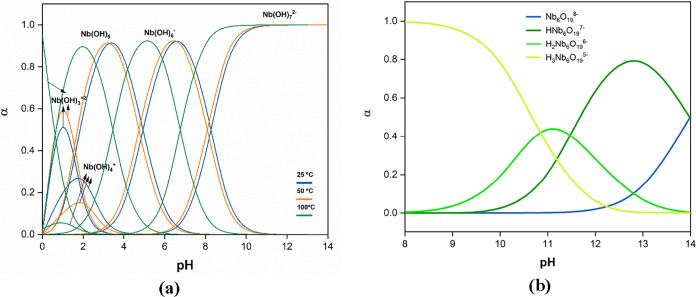
Speciation diagram of the Nb–H_2_O system by (a) PHREEQC-SIT databank at 25, 50, and 100 °C and (b) data from the literature for hexaniobate species in alkaline medium at 25 °C. Initial conditions: [Nb] = 1 mol/L and 1 atm.

According to the literature,^27,29^ the hexaniobate species are present in an alkaline medium. Figure 1b shows the distribution of species, and the major formation of hexaniobates occurs at pH > 8, and only the third protonation occurs at lower pH values.

The system K–Nb–H_2_O was modeled on OLI Studio and considered the formation of solid phases that may occur in the aqueous medium, as shown in Figure 2. It is noticeable that Nb_2_O_5_ is the dominant solid at lower pH values and that KNbO_3_ is formed with an increase in pH (pH > 4). However, the OLI database contains only stable-phase KNbO_3_, so the formation of metastable phases, such as K_4_Nb_6_O_17_, is not considered.

**Figure 2:**
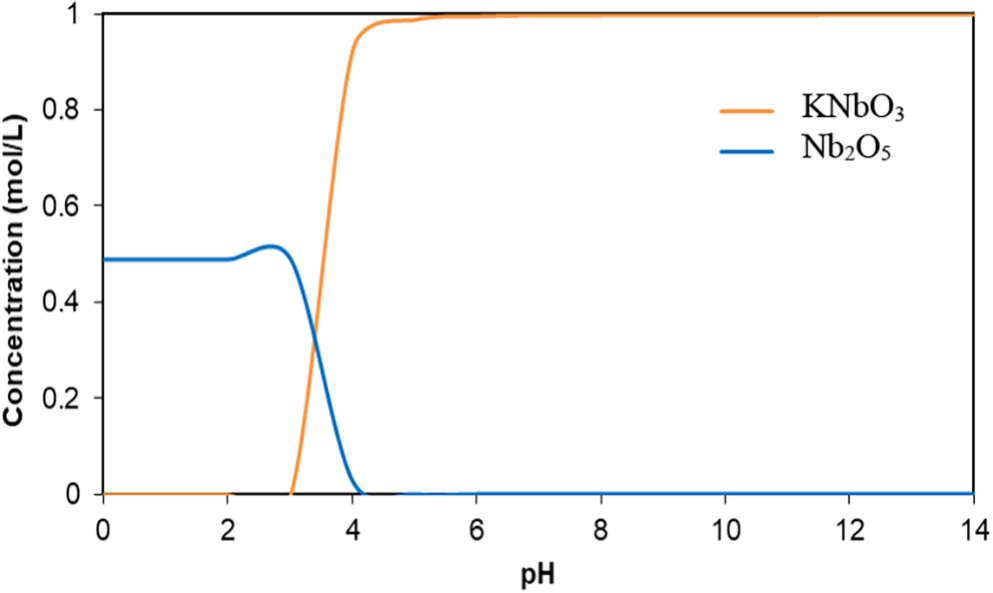
Speciation diagram of the K–Nb–H_2_O system at 25 °C and 1 atm. (Initial [KNbO_3_] = 1 mol/L – OLI Studio databank – AQ thermodynamic framework).

### 3.2 Kinetics of Niobic Acid Dissolution in an Alkaline Medium

The dissolution of niobic acid was monitored as a function of the concentration of KOH solution throughout 312 h at 30 ± 0.5 °C and 200 rpm, as shown in Figure 3. If all niobium could be dissolved in the KOH solution, the niobium concentration would be 0.15 (at 0.10 and 0.25 mol/L KOH solution) and 0.37 mol/L (at 0.50 mol/L KOH solution) (dashed gray line in the figures).

**Figure 3:**
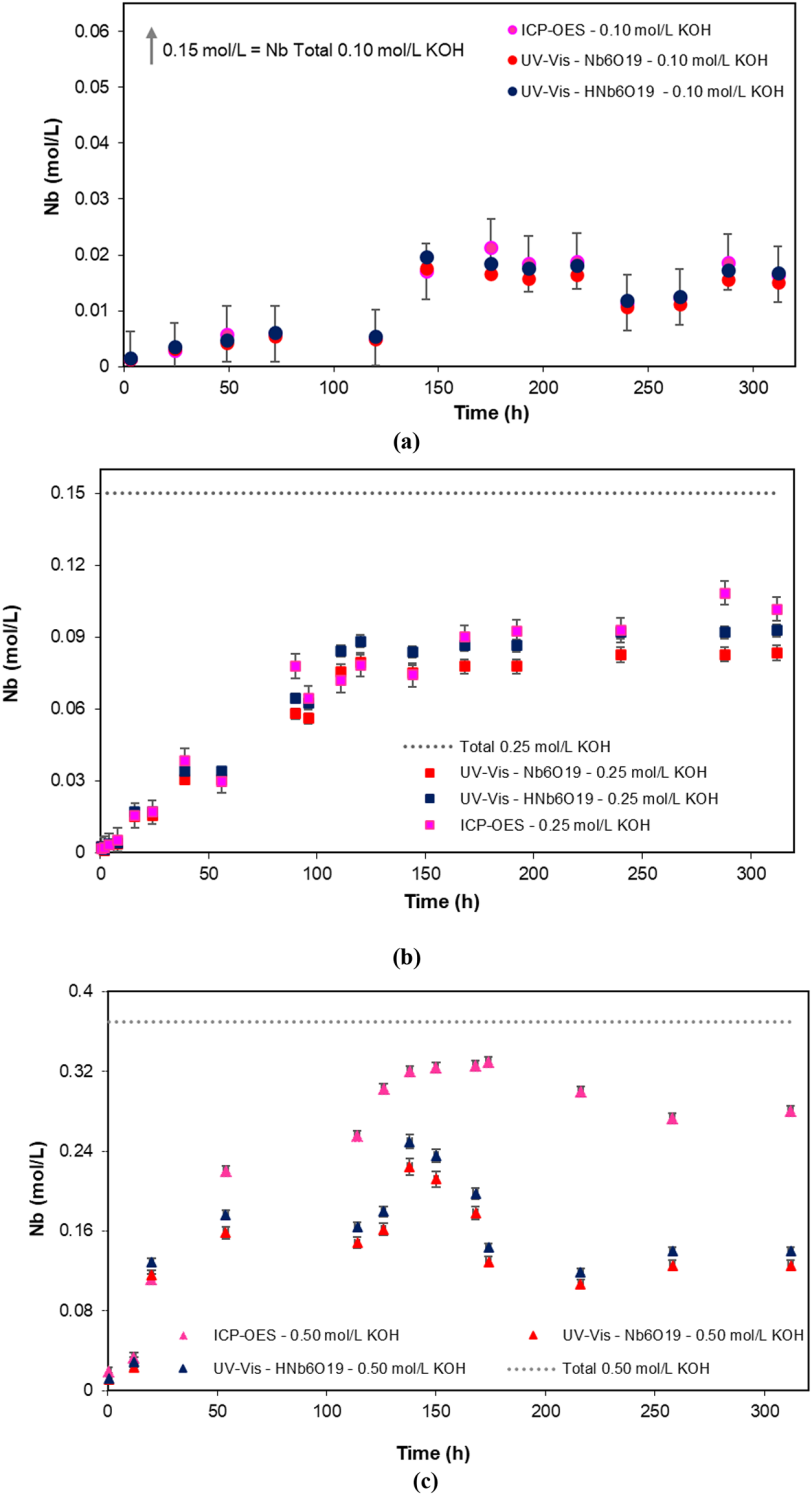
Aqueous niobium concentration measured by ICP-OES and UV spectroscopy (γ = 246.5 nm) from the equilibrium study of niobic acid in (a) 0.10, (b) 0.25, and (c) 0.50 mol/L KOH solutions at 30 ± 0.5 °C, 200 rpm, pH of 12.8 ± 0.4, 13.5 ± 0.1, and 13.8 ± 0.6, respectively (blue: HNb_6_O_19_^–7^; red: Nb_6_O_19_^–8^ by the UV–vis method; pink: Nb total by ICP-OES; gray arrow (a) and dashed line (b, c): maximum of niobium concentration).

The niobium content in the solution was measured by UV spectroscopy through the hexaniobate species Nb_6_O_19_^8–^ and HNb_6_O_19_^7–^ quantification. During the study of equilibrium, the pH value was above 12; therefore, the UV spectroscopy method could be effectively used.^24^ As mentioned before, in alkaline media, the niobium is complexed into hexaniobate, and the species Nb_6_O_19_^8–^ and HNb_6_O_19_^7–^ are dominant at higher pH values (Figure 1b). The pH behavior of niobic acid in KOH solutions with time is given in Table S1 (Supporting Information).

A difference in the niobium quantification between the ICP-OES and UV–vis methods is noted, and its variation increases with the increase in KOH concentration and, consequently, an increase in Nb dissolved in the solution. According to the method used in this work, based on the study of Deblonde et al.,^29^ the nature of the alkali ion alters the wavelength at which maximum absorbance is observed (246.5 and 247.5 in NaOH and KOH solutions, respectively). This occurs due to the ion-pairing effect between the cations (Cs > K > Na) and is reflected in the stabilization of the electron. According to the authors, the strong ion-pairing effect in the K^+^ media allows the stabilization of protonated clusters. The increasing hydroxide concentration promotes the deprotonation of hexaniobate species, favoring the formation of Nb_6_O_19_^8–^ cluster and influencing the formation of polyoxoniobates. However, an equilibrium between deprotonated and monoprotonated clusters is observed at 240 nm, close to the wavelength of Nb quantification. The hydroxide concentration also influences the system, showing that an increase in ionic strength decreases the equilibrium constant.

Additionally, some studies reported the presence of species such as H_*x*_Nb_7_O_22_^*x*–9^ as an intermediate from decaniobate Nb_10_O_28_^6–^ and monoprotonated H_*x*_Nb_6_O_19_^*x*–8^ at high pH values in the same region of hexaniobates.^27,45–47^ This may result in an underestimation of the total of aqueous niobium. It is necessary to account for the stability of polyoxoniobates as a limitation of the method, which may influence the measurements. In another study by our research group,^48^ the data of kinetics of dissolution of niobic acid were modeled in 0.10 and 0.25 mol/L KOH solutions using machine learning techniques. The random forest model was used to fit and compare the linear regression of the data. As a result, the ICP-OES data fit the proposed model better due to differences in the square error calculations of both methods: ICP-OES and UV–vis.

According to the OLI Studio simulation, the solubility of niobium oxide increases with the increase of KOH concentration in the aqueous medium. Experimentally, following OLI prediction, the increase in KOH concentration favors the dissolution of niobic acid. The concentration of aqueous Nb increases with time until the concentration of niobium in solution becomes constant (less than 10% variation, which is used as an operational condition for stabilization), indicating a quasi-equilibrium time after approximately 100 h of experiment in all tested conditions. However, it is possible to observe variations in Nb concentration quantified using the UV–vis method in the experiment with a higher KOH concentration of 0.50 mol/L between 140 and 170 min. This variation could be related to the kinetics of the formation of polyoxoniobates in solution, which directly influences the solubility of niobic acid. Even though Lindqvist ions have been known for a long time,^46^ the properties of polyoxometalate ions in different electrolytes are still scarce, especially for niobium. However, Deblonde et al^49^ showed that the presence of divalent cations such as Ca^2+^ significantly decreases the formation of hexaniobate species, highlighting the importance of electrolyte composition on Nb­(V) aqueous chemistry.

From this study, it was possible to infer the dissolution of niobic acid in the KOH solution in the mentioned conditions of 30 ± 0.5 °C and 200 rpm. At the end of each experiment, the niobium concentrations for each studied condition (0.10, 0.25, and 0.50 mol/L KOH) given by ICP-OES were 0.016, 0.101, and 0.286 mol/L and by the UV–vis method were 0.018, 0.102, and 0.154 mol/L, respectively.

The dependence of the dissolution of niobic acid on the KOH concentration and the fitted equation of the niobium concentration from the equilibrium study are presented in the Supporting Information (Figure S2 and Table S3, respectively). Niobic acid is known to be insoluble in water, especially at low pH. This result is in agreement with the literature, which reports an increase in the presence of Nb­(V) in an alkaline medium.^27,29,50,51^ The fitted equation of the niobium concentration from the equilibrium study is shown in Table S3.

### 3.3 Dissolution of Niobium Compounds in an Alkaline Medium: Experimental and Modeling

Thermodynamic modeling was also performed on OLI Studio to predict the solubility of niobic acid (Nb_2_O_5_·*n*H_2_O) and potassium niobate (KNbO_3_). The temperature and concentration of niobium in aqueous media, with and without the presence of electrolytes, were considered.

The maximum dissolved concentrations were experimentally measured with Crystal 16 to validate the simulations. It is important to emphasize that the measurements from Crystal 16 correspond to the dissolution of the solids (clear points) and therefore represent an indication of the true solubility under the defined experimental conditions. As the method relies on dissolution of the solids during the heating cycle, no solid residue remains at the end of the experiment. As a result, it was not possible to recover material from the experiments for crystallographic and/or structural characterization. Furthermore, it is important to emphasize that the results obtained experimentally and through simulations in OLI Studio cannot be effectively compared, since the experimental tests evaluated the solid dissolution behavior and not the thermodynamic solubility.

The measured concentrations were expressed in grams per liter. Since the potassium niobate used in this work is a mixture of phases (K_4_Nb_6_O_17_ and KNbO_3_) crystallized from an alkaline liquor^8^ (detailed composition of the solids in Supporting Information Table S2), the molar concentration cannot be calculated without introducing a large error associated with an arbitrary phase composition.

Figure 4 shows the dissolution of niobic acid in KOH solutions of different concentrations.

**Figure 4:**
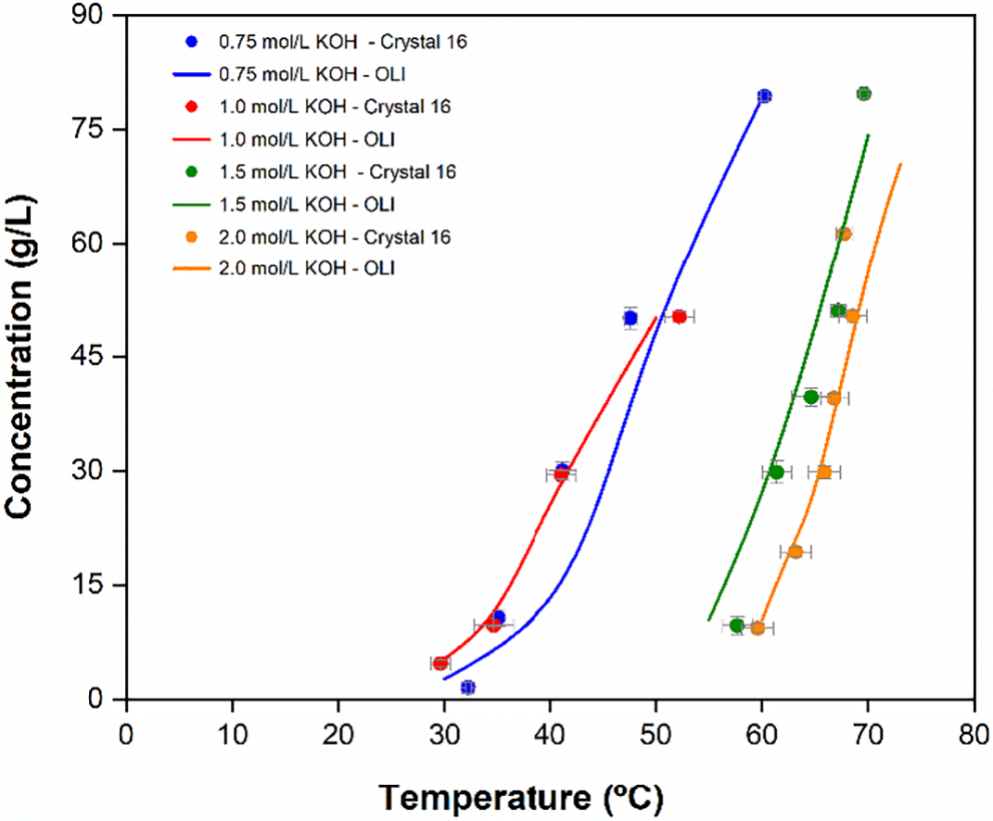
Concentration of niobic acid (Nb_2_O_5_·*n*H_2_O) in KOH versus temperature, obtained from Crystal 16 and predicted by OLI Studio.

OLI simulations and experimental results show a good fit, with an expressive increase in the dissolution of niobic acid with the temperature in the range of 20–70 °C and a slight decrease with the increase in the KOH concentration. The OLI Studio simulation shows the formation of potassium niobate as the stable phase (KNbO_3_) at concentrations higher than 1.5 mol/L (Figure S3). The Crystal 16 methodology adopted in this study was based on the dissolution of a known quantity of solids in a known amount of solvent with an increase in temperature. The data is collected when this solid is fully dissolved. Hence, no solid phase remained to be analyzed at the end of the experiment, since the maximum dissolution of the solid in the aqueous medium was considered. No evidence of formation of any solids from the reaction of dissolved Nb­(V) in a highly concentrated KOH solution was found. Even when the solution temperature was subsequently lowered to 20 °C after full dissolution at a very high cooling rate (−10 °C/min) and kept under stirring for over 48 h, no solids were seen, likely due to the slow kinetics of potassium niobate formation.

The solubility of niobic acid in distinct media reported in the literature in the past years (Table 2) was carried out under distinct conditions of the present work, so that the values shown in Table 2 and those determined in this work are not comparable.

For potassium niobate, the measurement was based on the temperature at which the solid was fully dissolved. Additionally, at the end of the experiment, no remaining solids were present in the vial for analysis. The values of the dissolution of potassium niobate evaluated in a range of 0–1.0 mol/L of the KOH solution are shown in Figure 5. To our knowledge, no experimental data have been reported on the solubility or dissolution of any potassium niobate phase in an alkaline medium. As an overall trend, the dissolution of crystallized potassium niobate increases with temperature. On the other hand, it decreases with an increase in KOH concentration, such that even the lowest concentration of KOH investigated (0.10 mol/L) lowers the dissolution by roughly 70%.

**Figure 5:**
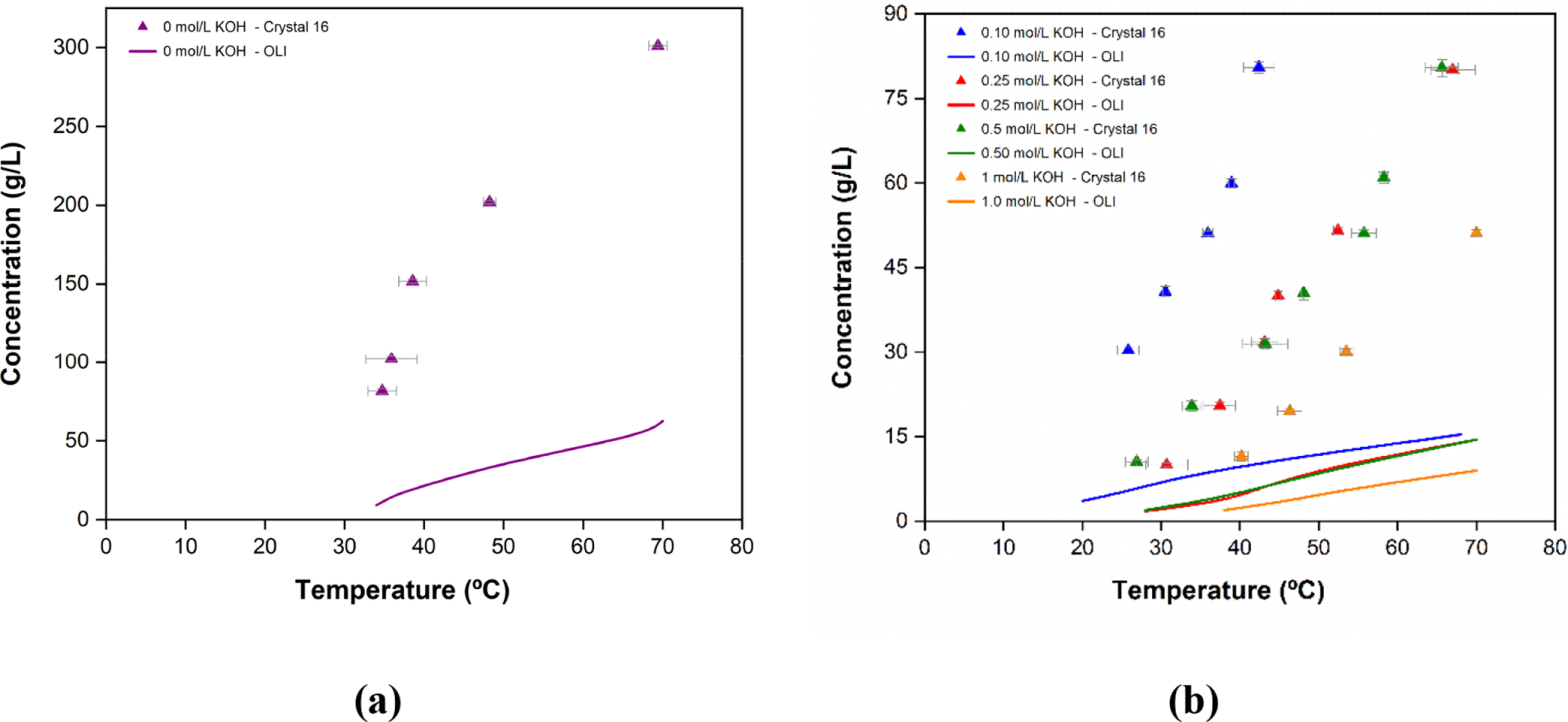
Dependence of the dissolution of crystallized potassium niobate in (a) water and (b) KOH on temperature obtained from Crystal 16 and predicted by OLI Studio stream analyzer.

According to the physicochemical analysis of the material, shown in the previous study (Souza et al.), two main potassium niobate crystals are present in the solids: the dominant and metastable phase K_4_Nb_6_O_17_ and the stable-phase KNbO_3_. This indicates that the crystallization of the stable phase may occur via a metastable phase in the first stage. Consequently, these phases could exhibit different dissolutions in an aqueous medium, and the measured values represent the overall dissolution behavior of the mixed-phase sample and cannot be interpreted as the thermodynamic solubility of an individual phase. Moreover, as the potassium niobate used in this study was produced by cooling crystallization from an alkaline liquor from the leaching of Fe–Nb alloy fines, the presence of impurities is expected, as presented in the chemical composition of KN reported in a previous study.^8^ The impurities may have a considerable effect on the dissolution of a system by combining and reacting chemically in the solution and forming complexes. In addition to these factors, nonequilibrium effects such as supersaturation and thermodynamic database may also contribute to these discrepancies.

Crystallized potassium niobate was shown to be more soluble than the stable phase when compared with the solubility results from OLI Studio. This is expected, since the potassium niobate used in the experiment is mainly formed of a metastable phase, usually more soluble than the stable phase of the K–Nb–H_2_O system.

The formation of hexaniobates species depends on the degree of alkalinity of the solution according to the suggested reactions shown in eqs 14–17. In dissolution, K_4_Nb_6_O_17_ first dissolves into the Nb_6_O_17_^4–^ complex and afterward transforms into the Nb_6_O_19_^8–^ hexaniobate ion. The same is observed in the KNbO_3_ phase, which first dissolves into NbO_6_^7–^ and then into the Lindqvist ion form.

14 $$K_{4}{\mathrm{Nb}}_{6}O_{17}={\mathrm{Nb}}_{6}{O_{17}}^{4-}+4K^{+}$$

15 $${\mathrm{Nb}}_{6}{O_{17}}^{4-}+4{\mathrm{OH}}^{-}={\mathrm{Nb}}_{6}{O_{19}}^{8-}+2H_{2}O$$

16 $${\mathrm{KNbO}}_{3}+6{\mathrm{OH}}^{-}={{{\mathrm{NbO}}_{6}}^{7}}^{-}+K^{+}+3H_{2}O$$

17 $$6{{\mathrm{NbO}}_{6}}^{7-}+17H_{2}O={\mathrm{Nb}}_{6}{O_{19}}^{8-}+34{\mathrm{OH}}^{-}$$

An effort was made to measure the solubility of KNbO_3_, hydrothermally synthesized, as the stable phase of the system, also using Crystal 16. However, due to the very slow kinetics of dissolution associated with the perovskite-type structure,^52^ it was not possible to determine the solubility by using the Crystal 16 approach, but only the dissolution behavior in the mentioned conditions. No changes were visible in the transmissivity, even after leaving the vials with very small concentrations of the material at 80 °C for 48 h.

It should be noted that the Crystal 16 experiments performed in this work provided only an apparent dissolution boundary under the specific dynamic heating/cooling protocol rather than strict equilibrium solubility data. This limitation is particularly relevant for systems containing phases with known slow dissolution kinetics, such as KNbO_3_. The applied heating rate may not allow sufficient time for complete equilibration, and the absence of visually detectable solids during cooling should not be interpreted as definitive evidence of the equilibrium dissolution. Hence, these results for the mixed-phase system are a qualitative indication of relative phase behavior between stable and metastable potassium niobate. Determination of true equilibrium solubility would likely require much longer equilibration times and independent solid-phase characterization, cross-verified with the final liquid composition after equilibration.

According to Deblonde et al.,^29^ the interaction between potassium and hexaniobate ions prevents the determination of solubility products of niobium in a potassium medium. These interactions may alter the free ion concentration and change the equilibrium of the solution. The formation of ion pairs has an important influence on the determination of solubility. Still, there is a lack of literature concerning the mechanisms of the formation of niobate complexes in aqueous solutions.

## 4 Conclusions

In this study, we have investigated the dissolution of niobic acid and potassium niobate under alkaline conditions experimentally and supported by the thermodynamic modeling from OLI Studio Stream Analyzer.

The use of UV–vis as a method of quantification of niobium in an equilibrium study of niobic acid shows that the hexaniobates may not be the only complexes formed during the reaction with KOH and that the complexation depends on the stability of polyoxoniobates in the solution. A change in the structure of niobic acid occurs after 100 h of reaction, and the presence of hexaniobate was confirmed by the characterization of the remaining solid.

The dissolution of niobic acid measured via Crystal 16 shows a slight decrease in the dissolution of the solid with an increase in KOH concentration from 0 to 2 mol/L at 20 to 70 °C. The strong interaction of K^+^ ions with hexaniobate ions may increase the dissolution of niobic acid up to a certain minimum concentration of KOH.

The dissolution of a mixed-phase potassium niobate was estimated and shown to increase with temperature but decrease with an increase of KOH concentration; for example, at 40 °C, the dissolution of potassium niobate is 11.5 g/L in 1 mol/L KOH and 80.5 g/L in 0.10 mol/L KOH. The presence of the metastable phase K_4_Nb_6_O_17_ suggests that the mixed phases exhibit a higher dissolution compared to the predictions of thermodynamic modeling for the stable-phase KNbO_3_.

The results from this study help address a gap in the literature on aqueous solubility and dissolution of niobium compounds under alkaline conditions and highlight the importance of solubility data on niobium for the development of a hydrometallurgical process.

## Supplementary Material



## Notes

FTIR and XRD of commercial niobic acid in a KOH solution and dissolution behavior, pH evolution during niobic acid dissolution experiments, chemical composition of niobic acid and potassium niobate, solubility of niobic acid and potassium niobate (KNbO_3_) from OLI Studio, fitted equations for niobium concentration as a function of time, and PHREEQC-SIT database outputs (PDF)
