# Supplementary material for: Dissolution Behavior of Niobium Compounds under Alkaline Conditions
Source: ACS Omega. 2026 Jul 20;11(30):45494–503. doi: 10.1021/acsomega.6c05225 (PMC13448943; doi:10.1021/acsomega.6c05225)
Supplement: Supplementary file 1 [file ao6c05225_si_001.pdf]

Supporting Information for

Dissolution behavior of niobium compounds under alkaline conditions

Cássia R. Souza [a], Frederico M. Penha [b*], Sônia D. F. Rocha [a]

[a] Department of Mining Engineering, School of Engineering, Universidade Federal de Minas Gerais (UFMG), Avenida Presidente Antônio Carlos, 6627, Belo Horizonte, Minas Gerais, Brazil.

[b] Department of Chemical Engineering, KTH Royal Institute of Technology (KTH), CBH School, Teknikrigen 42 11428, Stockholm, Sweden.

Table of Contents

Table S1. pH values of niobic acid in KOH solution during the equilibrium test.

Table S2. Chemical composition by XRF of niobic acid and potassium niobate.

Table S3. Fitted equations of solubility by equilibrium study and Crystal 16.

Figure S1. (a) FTIR and (b) XRD of niobic acid commercial and after 100 hours in the different concentrations of KOH solution at 30 ± 0.5 ºC.

Figure S2. Dissolution behaviour of niobic acid versus KOH concentration in solution from the equilibrium study at 30 ± 0.5 °C. Aqueous niobium concentration measured by ICP-OES.

Figure S3. Solubility of $Nb_2O_5nH_2O$ in KOH dependence on temperature. Initial condition: $[Nb_2O_5nH_2O]$ = 1 mol/L (OLI Studio databank – AQ thermodynamic framework).

Figure S4. Solubility of $KNbO_3$ as a function of temperature. Initial condition: $[KNbO_3]$ = 1 mol/L (OLI Studio databank – AQ thermodynamic framework).

Figure S5. Solubility of $KNbO_3$ in KOH dependence on temperature. Initial condition: [KNbO3] = 1 mol/L (OLI Studio databank – AQ thermodynamic framework).

FTIR and XRD of niobic acid at 100 hours of experiment (Figure S1), when the most of compound had partially dissolved in the KOH solution (Figures 3-5) shows a difference in bands 400 to 800 cm^{-1}, that indicates a change in the structure of the solids at 0.10 and 0.50 mol/L KOH when comparing with the niobic acid without previous contact with KOH solution. The niobic acid in 0.25 mol/L KOH shows a slight difference at 500 cm^{-1} with the same transmittance of niobic acid without contact in KOH solution. This behaviour may be related to a small interaction between the solid and the solution and can be corroborate by the small change in pH values during the experiment (see Table S2). The XRD also shows a change in the structure of amorphous niobic acid, with a reduction of the peaks.

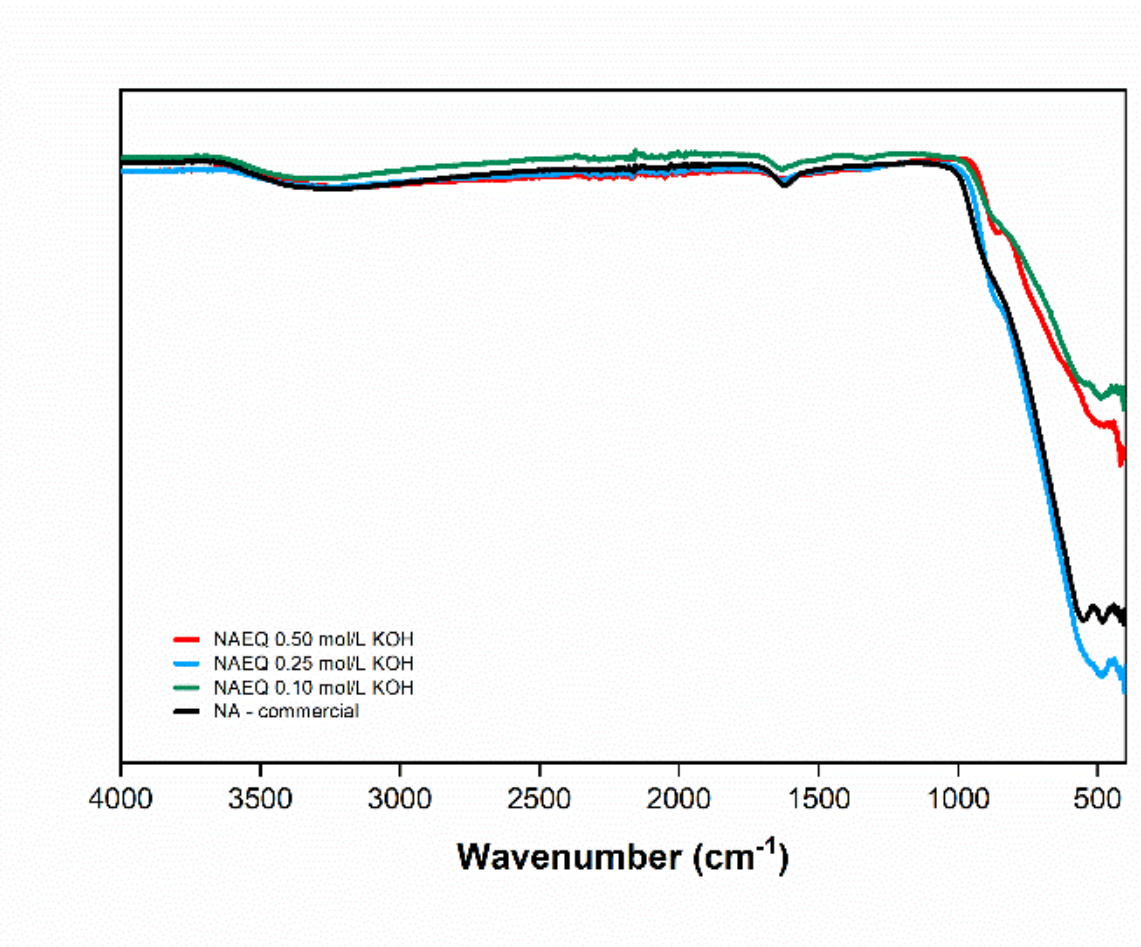

(a)

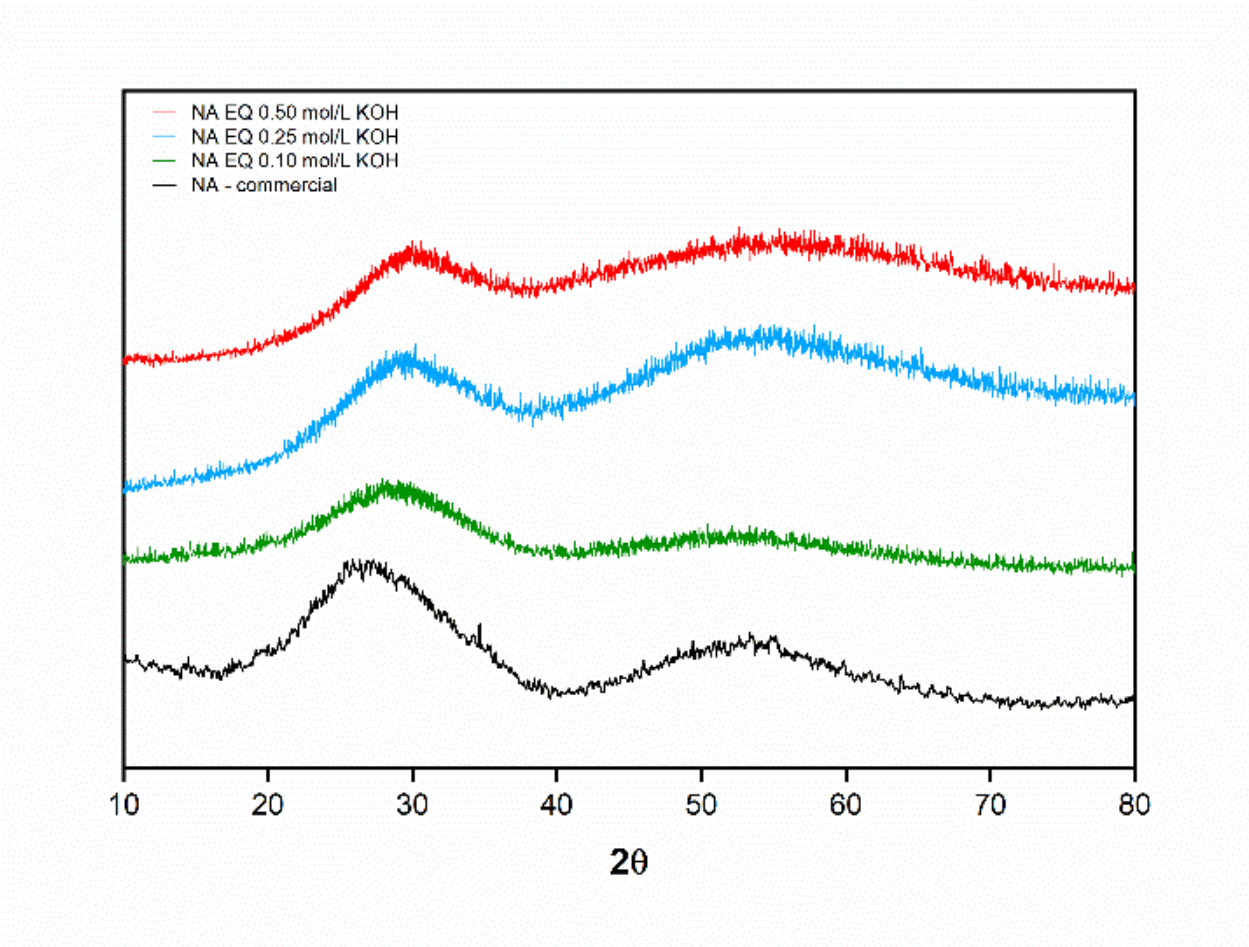

(b)

Figure S1. (a) FTIR and (b) XRD of niobic acid commercial and after 100 hours in the different concentrations of KOH solution at 30 ± 0.5 ºC.

Table S1 shows the pH behavior of niobic acid in KOH solution with time was monitored during the equilibrium study after the filtration of each sample. It is possible to observe that the pH values decreased during the experiment in each concentration of KOH. This may indicate a partial neutralization of the solution with niobic acid. At 0.50 mol/L, the highest concentration of KOH, the pH had a greater reduction in its value over time, which may suggest a greater interaction between KOH and niobic acid and more aqueous niobium available in solution.

Table S1. pH values of niobic acid in KOH solution during the equilibrium test.

**Time (h)**	**0.10**	**0.25**	**0.50**
		**(mol/L) KOH**	
		**pH**	
1	13.30	13.69	14.49
2	13.45	13.60	14.45
24	13.37	13.49	13.97
72	12.75	13.39	13.65
120	12.43	13.50	13.60
174	12.58	13.53	13.25
192	12.64	13.32	13.10
312	12.50	13.31	12.90

Figure S2 shows the dependence of solubility of niobic acid with the KOH concentration in the equilibrium study.

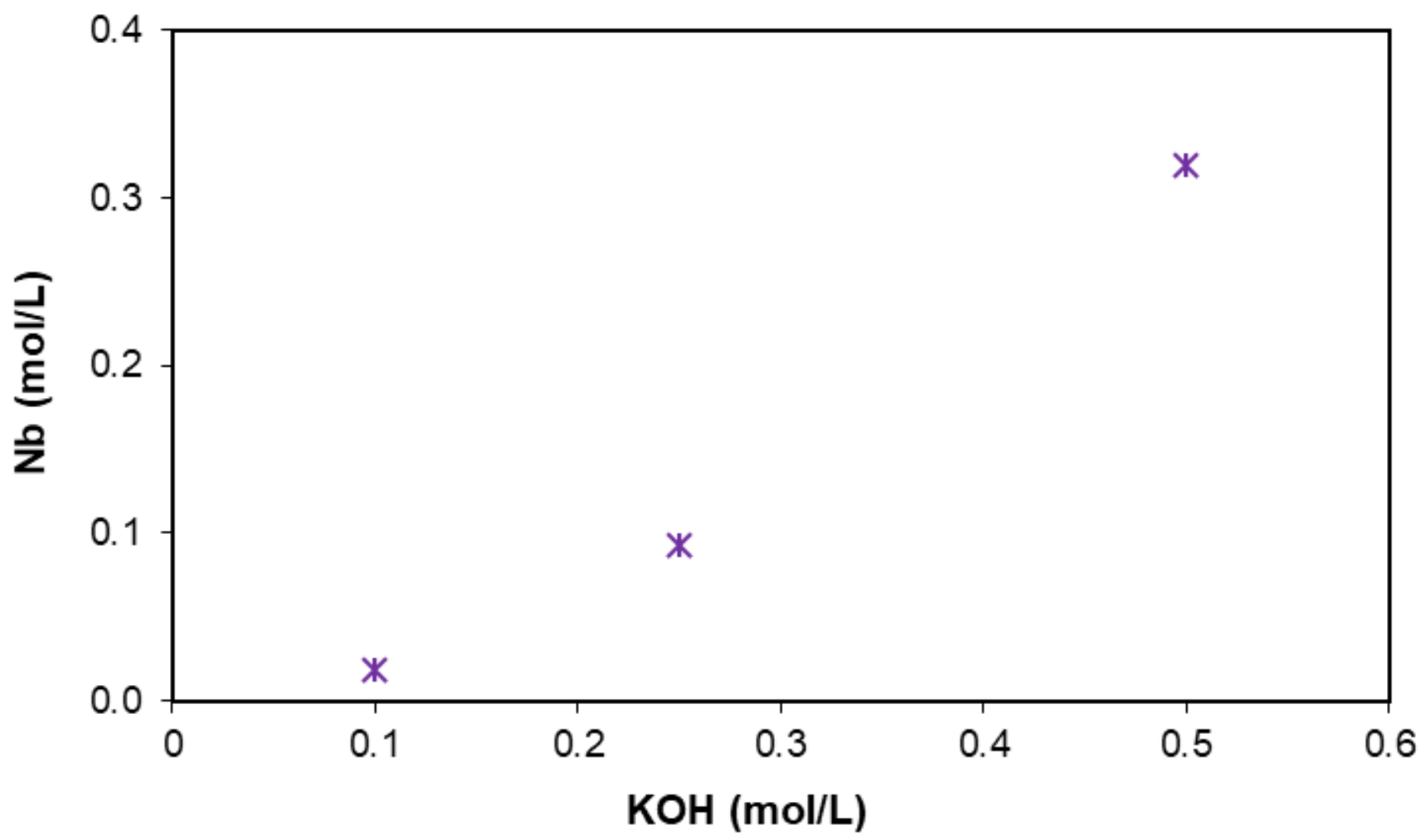

Figure S2. Dissolution behaviour of niobic acid versus KOH concentration in solution from the equilibrium study at 30 ± 0.5 ˚C. Aqueous niobium concentration measured by ICP-OES.

Table S2 shows the chemical composition of potassium niobate crystallized from an alkaline liquor Souza et al., 2024 – preprint) (mix of phases: $K_4Nb_6O_{17}$ and $KNbO_3$ and niobic acid from CBMM.

Table S2. Chemical composition by XRF of niobic acid and potassium niobate.

**Content (%)**	**Nb**	**O**	**K**	**Fe**	**S**	**Ta**	**Ca**	**Si**	**Al**
Potassium Niobate	44.26	24.4	26.22	0.24	0.04	<0.01	<0.01	<0.01	<0.01
Niobic Acid	69.36	30.05	-	-	-	0.08	0.09	-	-

Solubility of niobic acid in a range of 0 to 5 mol/L of KOH at different temperatures was measured (Figure S3). For this, the formation of other phases was allowed during the thermodynamic modeling of solubility of niobic acid, due to possible reactions with KOH. From 1.5 mol/L KOH Nb is consumed from the solution and react with potassium, forming potassium niobate as $KNbO_3$.

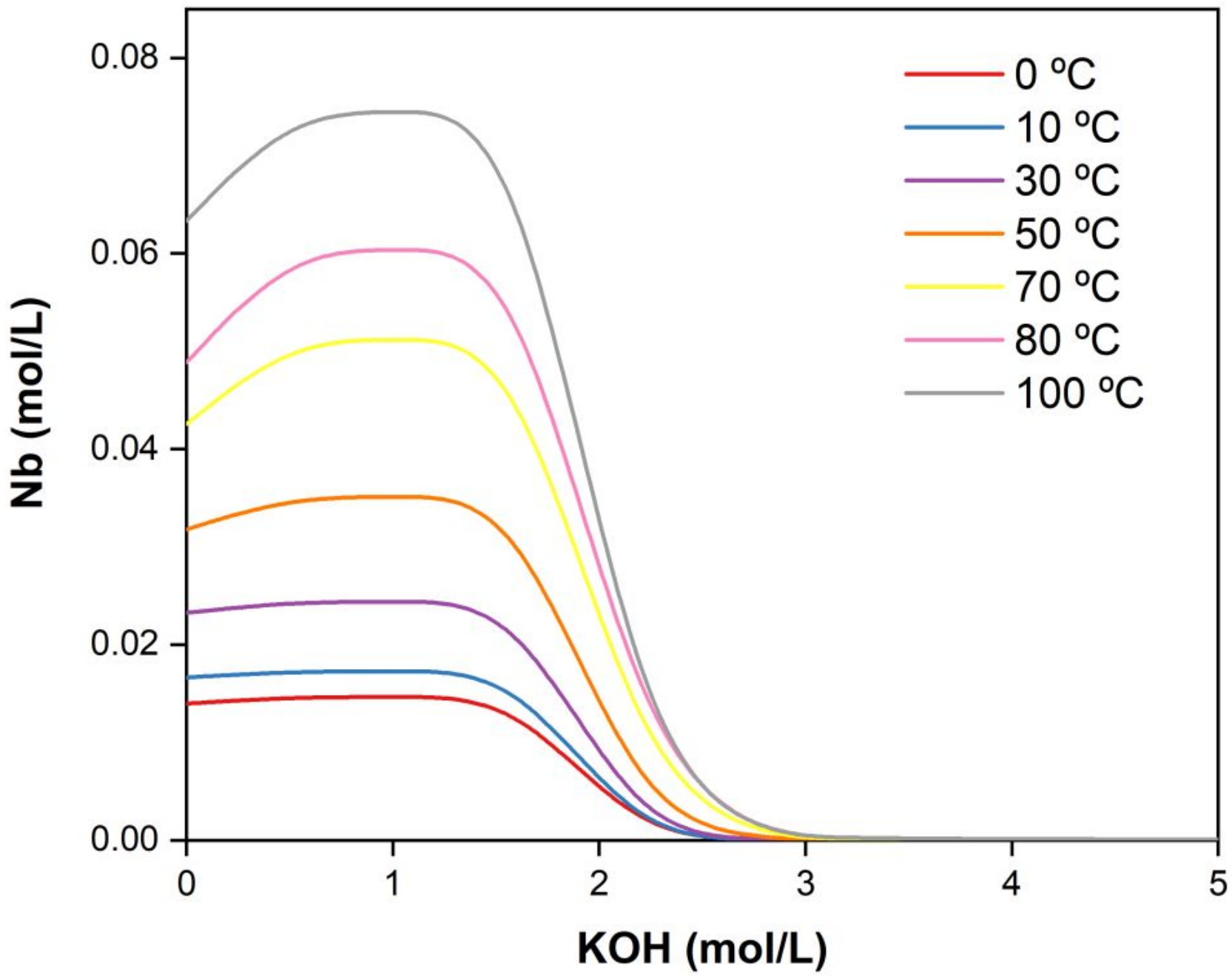

Figure S3. Solubility of $Nb_2O_5nH_2O$ in KOH dependence on temperature. Initial condition: $[Nb_2O_5nH_2O]$ = 1 mol/L (OLI Studio databank – AQ thermodynamic framework).

According to the OLI Studio modeling, the solubility of the $KNbO_3$ phase in water is not linear with the temperature, as shown in Figure S3. The solubility exhibits a slight increase until 40ºC, followed by a rapid increase until reaching 90 ºC, the highest solubility point. Beyond this temperature, the solubility of potassium niobate decreases. The influence of the temperature and KOH concentration on the solubility of $KNbO_3$ given by OLI Studio can be seen in Figure S4.

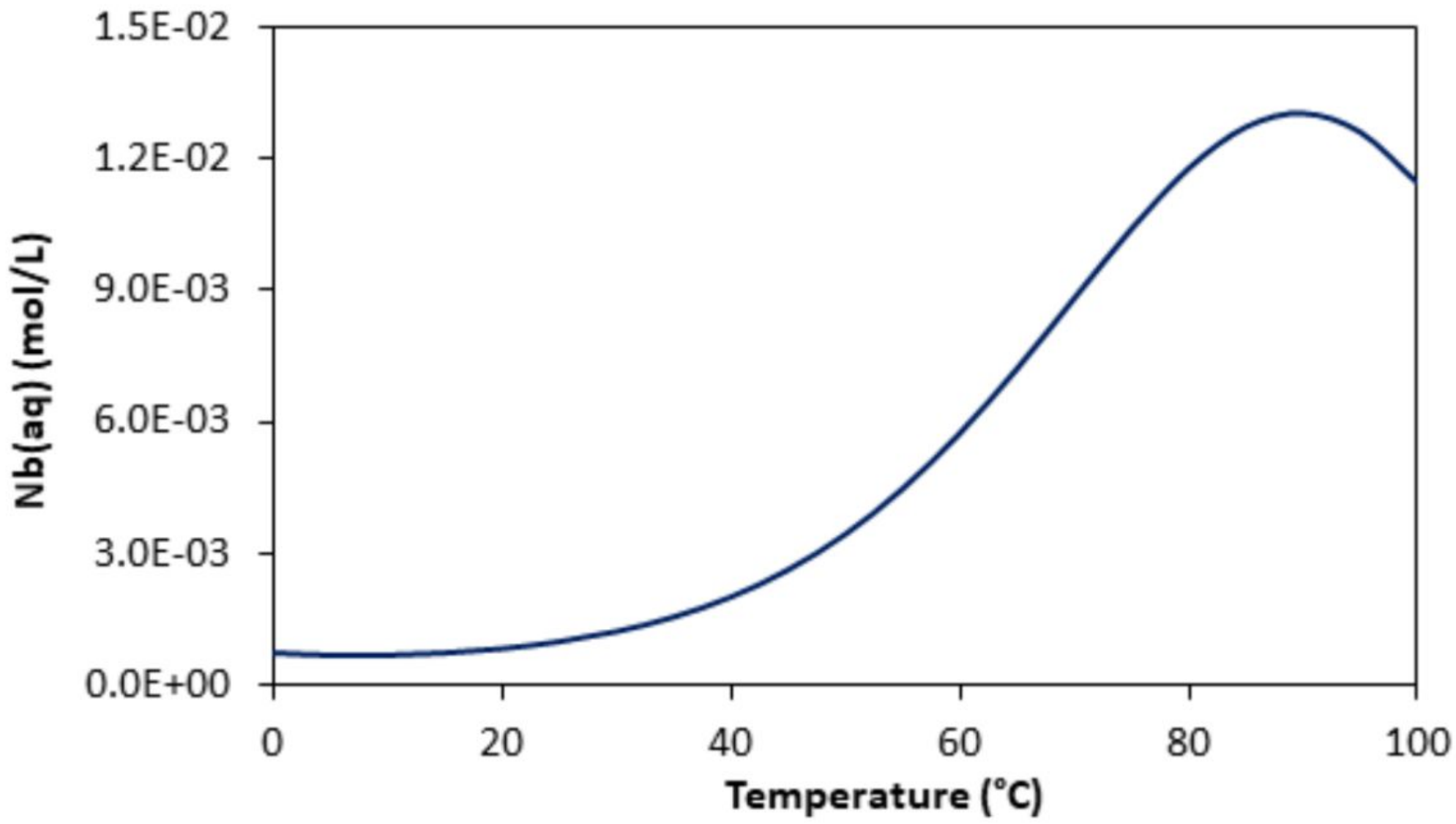

Figure S4. Solubility of $KNbO_3$ as a function of temperature. Initial condition: [$KNbO_3$] = 1 mol/L (OLI Studio databank – AQ thermodynamic framework).

The solubility of $KNbO_3$ is strongly affected by the temperature and solution concentration. The solubility of potassium niobate decreases with the increasing in KOH concentration. However, the solubility increases with the temperature when the KOH concentration is lower.

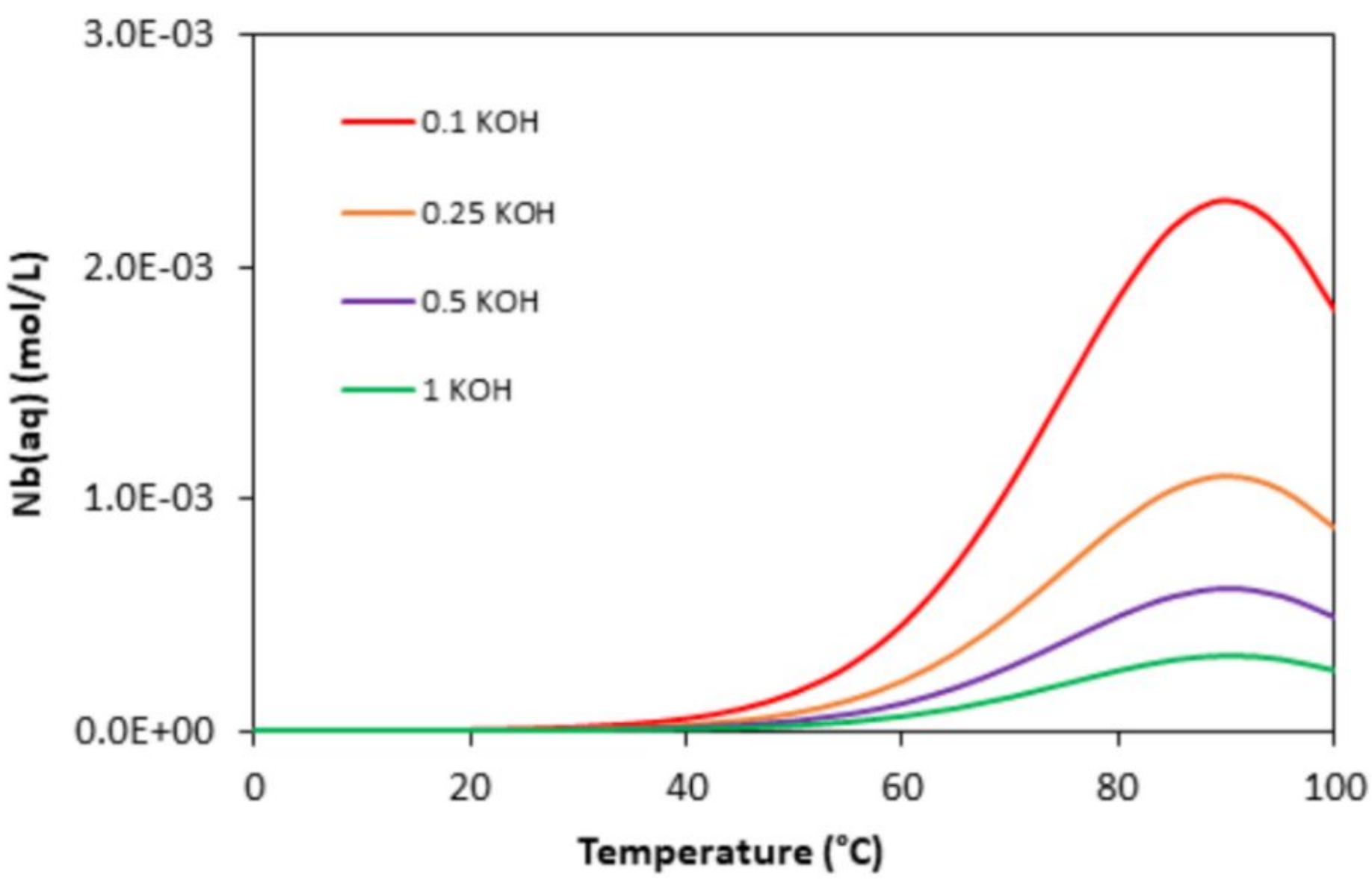

Figure S5. Solubility of $KNbO_3$ in KOH dependence on temperature. Initial condition: [KNbO3] = 1 mol/L (OLI Studio databank – AQ thermodynamic framework).

Table S3. Niobium concentration in the equilibrium study and Crystal 16.

Condition	Fitted Equation	R^2
	Equilibrium study (commercial niobic acid)	
0.10 - 0.50 mol/L KOH	C (g/L) =0.77±0.11*T (°C) –0.07±0.04	0.9590
	Potassium Niobate (mixed phases)	
0 mol/L KOH	C (g/L) = 5.56±0.60*T (°C) – 90.92±3.30	0.9556
0.10 mol/L KOH	C (g/L) = 2.43±0.34*T (°C) – 33.55±11.06	0.9273
0.25 mol/L KOH	C (g/L) = 1.93±0.05*T (°C) – 49.73±2.02	0.9969
0.50 mol/L KOH	C (g/L) = 1.52±0.09*T (°C) – 31.05±3.66	0.9786
1.00 mol/L KOH	C (g/L) = 1.34±0.03*T (°C) – 42.02±1.74	0.9982
	Niobic Acid (commercial)	
0.75 mol/L KOH	C (g/L) = 2.76±0.08*T (°C) – 86.09±3.78	0.9968
1.00 mol/L KOH	C (g/L) = 2.27±0.40*T (°C) – 68.33±3.21	0.9821
1.50 mol/L KOH	C (g/L) = 5.90±0.92*T (°C) – 335.94±6.16	0.9085
2.00 mol/L KOH	C (g/L) = 5.65±1.20*T (°C) – 332.06±7.80	0.8104

THERMODYNAMIC MODELING – PHREEQC SIT DATABASE

Nb in aqueous media

```
# Thermodynamic database ANDRA/RWM - THERMOCHIMIE-TDB (www.thermochimie-tdb.com)
# Version 9b0
# Nom : ThermoChimie project
# BDD Date: 10/8/2015
```

Comment: no comment

NAMED_EXPRESSIONS

#
formation of O2 from H2O
2H2O = O2 + 4H+ + 4e-
#
Log_K_O2
log_k -85.980
delta_H 559.526 kJ/mol
#
-analytic 1.20446E+1 0E+0 -2.9226E+4 0E+0 0E+0

SOLUTION_MASTER_SPECIES

#element species alk gfw_formula element_gfw
E e- 0 0 0
H H+ -1 H 1.0079
H(+1) H+ -1 H 1.0079
H(0) H2 0 H 1.0079
K K+ 0 K 39.0983
Nb Nb(OH)6- 0 Nb 92.9064
O H2O 0 O 15.999
O(0) O2 0 O 15.9994
O(-2) H2O 0 O 15.9994
Ox Ox-2 0 Ox 88.0196
S SO4-2 0 SO4 32.066
S(+2) S2O3-2 0 S 32.066
S(+3) S2O4-2 0 S2O4-2 128.128
S(+4) SO3-2 0 S 32.066
S(+6) SO4-2 0 SO4 32.066
S(-2) HS- 1 S 32.066
SIT
-epsilon

(NpO2)2(OH)2+2 Cl- -0.248
(NpO2)2(OH)2+2 ClO4- 0.57
(NpO2)2CO3(OH)3- Na+ 0
(NpO2)3(CO3)6-6 Na+ -0.46
(NpO2)3(OH)5+ Cl- -0.226
(NpO2)3(OH)5+ ClO4- 0.45
(PuO2)2(OH)2+2 Cl- -0.248
(PuO2)3(CO3)6-6 Na+ -0.46
(UO2)2(CO3)(OH)3- Na+ 0
(UO2)2(NpO2)(CO3)6-6 Na+ 0.09
(UO2)2(OH)2+2 Cl- 0.69
(UO2)2(OH)2+2 ClO4- 0.57
(UO2)2(OH)2+2 NO3- 0.49
(UO2)3(CO3)6-6 Na+ 0.37
(UO2)3(OH)4+2 Cl- 0.5
(UO2)3(OH)5+ Cl- 0.81
Ag(SeO3)- Na+ -0.024
Ag+ Cl- 0.031
Al(OH)+2 Cl- 0.09
Al(OH)2+ Cl- 0.09
Al+3 Cl- 0.33
Am(CO3)+ Cl- 0.129
Am(CO3)+ ClO4- 0.17
Am(CO3)2- Na+ -0.14
Am(CO3)3-3 Na+ -0.23
Am(CO3)5-6 Na+ -0.3
Am(Edta)- Na+ 0.01
Am(H2PO4)+2 Cl- 0.191
Am(H2PO4)+2 ClO4- 0.39
Am(NO3)+2 Cl- 0.191
Am(NO3)+2 ClO4- 0.39
Am(OH)+2 Cl- -0.04
Am(OH)+2 ClO4- 0.39
Am(OH)2+ Cl- -0.29
Am(OH)2+ ClO4- 0.17
Am(Ox)+ ClO4- 0.08
Am(Ox)2- Na+ -0.21
Am(Ox)3-3 Na+ -0.23
Am(SO4)+ Cl- 0.157
Am(SO4)+ ClO4- 0.22
Am(SO4)2- Na+ -0.05
Am+3 Cl- 0.23
Am+3 ClO4- 0.49
AmCl+2 Cl- 0.191
AmCl+2 ClO4- 0.39
AmCl2+ Cl- 0.129
AmF+2 Cl- 0.191
AmF+2 ClO4- 0.39
AmF2+ Cl- 0.129
AmF2+ ClO4- 0.17
AmO2(CO3)- Na+ -0.18
AmO2(CO3)2-3 Na+ -0.33
AmO2(CO3)3-5 Na+ -0.53
AmO2+ Cl- 0.09
AmO2+2 Cl- 0.39
B(OH)4- Na+ -0.07
Ba+2 Cl- 0.07
Ba+2 ClO4- 0.15
Ba+2 NO3- -0.28
Br- Na+ 0.05
CO3-2 Na+ -0.08
Ca+2 Cl- 0.14
Ca+2 ClO4- 0.27
Ca+2 NO3- 0.02
Ca4Th(OH)8+4 Cl- -0.01
Ca4Th(OH)8+4 ClO4- 0.21
Cd(HCO3)+ Cl- 0.2
Cd+2 Cl- 0.16
Cd+2 NO3- 0.09
CdCl+ Cl- 0.127
CdCl+ ClO4- 0.25
CdI+ Cl- 0.136
CdI+ ClO4- 0.27
Cit-3 Na+ -0.076
Cl- Na+ 0.03
Cm(CO3)2- Na+ 0.34
Cm(CO3)3-3 Na+ 0.16
Cm(H2PO4)+2 Cl- 0.191
Cm(H2PO4)+2 ClO4- 0.39
Cm(OH)+2 Cl- -0.04

Cm(OH)+2 ClO4- 0.39
Cm(OH)2+ Cl- -0.27
Cm(OH)2+ ClO4- 0.17
Cm(SO4)2- Na+ -0.05
Cm+3 Cl- 0.23
Cm+3 ClO4- 0.49
CmCO3+ Cl- 0.35
CmCl+2 Cl- 0.191
CmCl+2 ClO4- 0.39
CmCl2+ Cl- 0.129
CmF+2 ClO4- 0.39
CmF2+ ClO4- 0.17
CmNO3+2 ClO4- 0.39
CmSO4+ Cl- 0.157
CmSO4+ ClO4- 0.22
Cn- Na+ 0.07
Co+2 Cl- 0.16
Co+2 ClO4- 0.34
Co+2 NO3- 0.14
Cr+3 Cl- 0.3
Cr+3 NO3- 0.27
Cu+ ClO4- 0.11
Cu+2 Cl- 0.08
Cu+2 ClO4- 0.32
Cu+2 NO3- 0.11
Edta-4 Na+ 0.32
Eu(CO3)+ Cl- 0.129
Eu(CO3)+ ClO4- 0.17
Eu(CO3)2- Na+ -0.14
Eu(CO3)3-3 Na+ -0.23
Eu(H2PO4)+2 Cl- 0.191
Eu(H2PO4)+2 ClO4- 0.39
Eu(NO3)+2 Cl- 0.191
Eu(NO3)+2 ClO4- 0.39
Eu(OH)+2 Cl- -0.04
Eu(OH)+2 ClO4- 0.39
Eu(OH)2+ Cl- -0.29
Eu(OH)2+ ClO4- 0.17
Eu(SO4)+ Cl- 0.157
Eu(SO4)+ ClO4- 0.22
Eu(SO4)2- Na+ -0.05
Eu+3 Cl- 0.23
Eu+3 ClO4- 0.49
EuCl+2 Cl- 0.191
EuCl+2 ClO4- 0.39
EuCl2+ Cl- 0.129
EuF+2 Cl- 0.191
EuF2+ Cl- 0.129
F- Na+ 0.02
Fe(OH)+2 Cl- 0.186
Fe+3 ClO4- 0.56
Fe+3 NO3- 0.42
H(Cit)-2 Na+ -0.04
H(Edta)-3 Na+ -0.1
H(Ox)- Na+ -0.07
H(SO4)- Na+ -0.01
H(SeO3)- Na+ 0.02
H(SeO4)- Na+ 0
H+ Cl- 0.12
H+ ClO4- 0.14
H+ NO3- 0.07
H2(Cit)- Na+ -0.05
H2(Edta)-2 Na+ -0.37
H2(PO4)- Na+ -0.08
H2(SiO4)-2 Na+ -0.1
H3(Edta)- Na+ -0.33
H3(SiO4)- Na+ -0.08
H5(Edta)+ Cl- -0.23
H5(Edta)+ ClO4- -0.23
H5(Edta)+ NO3- -0.23
H6(Edta)+2 Cl- -0.2
H6(Edta)+2 ClO4- -0.2
H6(Edta)+2 NO3- -0.2
HCO3- Na+ 0
HGlu- Na+ -0.07
HIsa- Na+ -0.07
HMoO4- Na+ -0.099
HPO4-2 Na+ -0.15
Hf(NO3)2+2 ClO4- 0.84
Hf(OH)+3 ClO4- 0.57
Hf+4 Cl- 0.89
Hf+4 ClO4- 0.89
Hf+4 NO3- 0.89
HfCl+3 ClO4- 0.87
HfCl2+2 ClO4- 0.84
HfF+3 ClO4- 0.63
HfF2+2 ClO4- 0.47
HfNO3+3 ClO4- 0.91
Hg(SeO3)2-2 Na+ 0.008
Hg+2 Cl- 0.168
Hg2+2 Cl- 0.054
Ho(CO3)+ Cl- 0.129
Ho(CO3)+ ClO4- 0.17
Ho(CO3)2- Na+ -0.14
Ho(CO3)3-3 Na+ -0.23
Ho(H2PO4)+2 Cl- 0.191
Ho(H2PO4)+2 ClO4- 0.39
Ho(NO3)+2 Cl- 0.191
Ho(NO3)+2 ClO4- 0.39
Ho(OH)+2 Cl- -0.04
Ho(OH)+2 ClO4- 0.39
Ho(OH)2+ Cl- -0.29
Ho(OH)2+ ClO4- 0.17
Ho(SO4)+ Cl- 0.157
Ho(SO4)+ ClO4- 0.22
Ho(SO4)2- Na+ -0.05
Ho+3 Cl- 0.23
Ho+3 ClO4- 0.49
HoCl+2 Cl- 0.191
HoCl+2 ClO4- 0.39
HoCl2+ Cl- 0.129
HoF+2 Cl- 0.191
HoF+2 ClO4- 0.39
HoF2+ Cl- 0.129
HoF2+ ClO4- 0.17
I- Na+ 0.08
IO3- Na+ -0.06
K+ Cl- 0
K+ NO3- -0.11
Li+ Cl- 0.1
Li+ ClO4- 0.15
Li+ NO3- 0.08
Mg(Cit)- Na+ 0.03
Mg(Edta)-2 Na+ -0.01
Mg(HEdta)- Na+ 0.11
Mg(Ox)2-2 Na+ -0.15
Mg+2 Cl- 0.19
Mg+2 ClO4- 0.33
Mg+2 NO3- 0.17
Mn+2 Cl- 0.13
Mo7O21(OH)3-3 Na+ -1.455
Mo7O22(OH)2-4 Na+ -1.346
Mo7O23(OH)-5 Na+ -1.145
Mo7O24-6 Na+ -1.265
MoO4-2 Na+ 0.034
NH4+ Cl- -0.01
NH4+ ClO4- -0.08
NH4+ NO3- -0.06
NO3- Na+ -0.04
Na+ ClO4- 0.01
Ni(CO3)2-2 Na+ 0.003
Ni(Cit)- Na+ 0.22
Ni(Cn)4-2 Na+ 0.185
Ni(Cn)5-3 Na+ 0.25
Ni(HCO3)+ Cl- 0.085
Ni(NO3)+ Cl- 0.06
Ni(NO3)+ ClO4- 0.44
Ni(OH)+ Cl- -0.01
Ni(OH)+ ClO4- 0.14
Ni(OH)3- Na+ 0.88
Ni(SO4)2-2 Na+ -0.263
Ni(Scn)+ Cl- 0.11
Ni(Scn)+ ClO4- 0.31
Ni(Scn)3- Na+ 0.66
Ni+2 Cl- 0.17
Ni+2 ClO4- 0.37
Ni+2 NO3- 0.182
Ni4(OH)4+4 ClO4- 1.08
NiCl+ Cl- 0.1
NiCl+ ClO4- 0.34
NiF+ Cl- 0.065
NiF+ ClO4- 0.34
NiHS+ ClO4- -0.85
Np(CO3)(OH)3- Na+ -0.11
Np(CO3)+ Cl- 0.129
Np(CO3)+ ClO4- 0.17
Np(CO3)2- Na+ -0.14
Np(CO3)3-3 Na+ -0.23
Np(CO3)4-4 Na+ -0.09
Np(CO3)5-6 Na+ 0
Np(NO3)+3 ClO4- 0.71
Np(OH)+2 Cl- -0.04
Np(OH)+2 ClO4- 0.39
Np(OH)+3 Cl- 0.2
Np(OH)+3 ClO4- 0.5
Np(OH)2+ Cl- -0.29
Np(OH)2+ ClO4- 0.17
Np(OH)2+2 Cl- 0.1
Np(OH)3+ Cl- 0.05
Np(OH)4(CO3)-2 Na+ -0.13
Np(SO4)+ Cl- 0.157
Np(SO4)+ ClO4- 0.22
Np(SO4)+2 Cl- 0.232

Np(SO4)+2 ClO4- 0.48
Np(SO4)2- Na+ -0.05
Np+3 Cl- 0.23
Np+3 ClO4- 0.49
Np+4 Cl- 0.4
Np+4 ClO4- 0.84
NpCl+3 ClO4- 0.81
NpF+3 ClO4- 0.58
NpF2+2 Cl- 0.186
NpF2+2 ClO4- 0.38
NpF3+ Cl- 0.1
NpI+3 ClO4- 0.77
NpO2(CO3)- Na+ -0.18
NpO2(CO3)2-2 Na+ -0.02
NpO2(CO3)2-3 Na+ -0.33
NpO2(CO3)2OH-4 Na+ -0.4
NpO2(CO3)3-4 Na+ -0.4
NpO2(CO3)3-5 Na+ -0.53
NpO2(Cit)-2 Na+ -0.06
NpO2(Edta)-3 Na+ 0.2
NpO2(HEdta)-2 Na+ 0.07
NpO2(HPO4)2-2 Na+ -0.1
NpO2(NO3)+ Cl- 0.22
NpO2(NO3)+ ClO4- 0.33
NpO2(OH)+ Cl- -0.003
NpO2(OH)+ ClO4- -0.06
NpO2(OH)2- Na+ -0.01
NpO2(Ox)- Na+ -0.4
NpO2(Ox)2-3 Na+ -0.3
NpO2(SO4)- Na+ -0.74
NpO2(SO4)2-2 Na+ -0.12
NpO2+ Cl- 0.09
NpO2+ ClO4- 0.25
NpO2+2 Cl- 0.223
NpO2+2 ClO4- 0.46
NpO2Cl+ Cl- 0.318
NpO2Cl+ ClO4- 0.5
NpO2F+ Cl- 0.197
NpO2F+ ClO4- 0.29
OH- Na+ 0.04
Ox-2 Na+ -0.08
PO4-3 Na+ -0.25
Pa+4 Cl- 0.3
PaO(OH)+2 Cl- 0.13
Pb(OH)3- Na+ 0.02
Pb(SO4)2-2 Na+ -0.52
Pb+2 Cl- 0.081
Pb+2 ClO4- 0.15
Pb+2 NO3- -0.2
PbF+ Cl- 0.14
Pd(SO4)2-2 Na+ -0.12
Pd+2 Cl- 0.16
PdBr+ Cl- 0.175
PdBr3- Na+ 0
PdBr4-2 Na+ 0.1
PdCl+ Cl- 0.175
PdCl3- Na+ 0
PdCl4-2 Na+ 0.1
Pu(CO3)+ Cl- 0.129
Pu(CO3)+ ClO4- 0.17
Pu(CO3)2- Na+ -0.14
Pu(CO3)3-3 Na+ -0.23
Pu(CO3)4-4 Na+ -0.09
Pu(CO3)5-6 Na+ 0
Pu(NO3)+2 Cl- 0.191
Pu(NO3)+2 ClO4- 0.39
Pu(NO3)+3 ClO4- 0.59
Pu(OH)+2 Cl- -0.04
Pu(OH)+2 ClO4- 0.39
Pu(OH)+3 Cl- 0.2
Pu(OH)+3 ClO4- 0.5
Pu(OH)2+ Cl- -0.29
Pu(OH)2+ ClO4- 0.17
Pu(OH)2+2 Cl- 0.1
Pu(OH)3+ Cl- 0.05
Pu(SO4)+ Cl- 0.157
Pu(SO4)+ ClO4- 0.22
Pu(SO4)+2 Cl- 0.232
Pu(SO4)+2 ClO4- 0.36
Pu(SO4)2- Na+ -0.05
Pu+3 Cl- 0.23
Pu+3 ClO4- 0.49
Pu+4 Cl- 0.37
Pu+4 ClO4- 0.82
PuBr+3 Cl- 0.1
PuBr+3 ClO4- 0.58
PuCl+3 Cl- 0.1
PuCl+3 ClO4- 0.85
PuF+3 Cl- 0.1
PuF+3 ClO4- 0.56
PuF2+2 Cl- 0.177
PuF2+2 ClO4- 0.36
PuF3+ Cl- 0.1
PuI+2 Cl- 0.191
PuI+2 ClO4- 0.39
PuI+3 Cl- 0.1
PuO2(CO3)2-2 Na+ -0.7
PuO2(CO3)3-4 Na+ -0.2
PuO2(CO3)3-5 Na+ -0.53
PuO2(OH)+ Cl- -0.003
PuO2(SO4)- Na+ -0.74
PuO2(SO4)2-2 Na+ -0.12
PuO2+ Cl- 0.129
PuO2+ ClO4- 0.24
PuO2+2 Cl- 0.223
PuO2+2 ClO4- 0.46
PuO2CO3- Na+ -0.18
PuO2Cl+ Cl- 0.318
PuO2Cl+ ClO4- 0.5
PuO2F+ Cl- 0.197
PuO2F+ ClO4- 0.29
PuO2F3- Na+ 0
PuO2NO3+ Cl- 0.22
PuO2NO3+ ClO4- 0.33
SO3-2 Na+ -0.08
SO4-2 Na+ -0.12
Scn- Na+ 0.05
SeO3-2 Na+ -0.08
SeO4-2 Na+ -0.12
Si2O2(OH)5- Na+ -0.08
Si2O3(OH)4-2 Na+ -0.15
Si3O5(OH)5-3 Na+ -0.25
Si3O6(OH)3-3 Na+ -0.25
Si4O8(OH)4-4 Na+ -0.26
Sm(CO3)+ Cl- 0.129
Sm(CO3)+ ClO4- 0.17
Sm(CO3)2- Na+ -0.14
Sm(CO3)3-3 Na+ -0.23
Sm(H2PO4)+2 Cl- 0.191
Sm(H2PO4)+2 ClO4- 0.39
Sm(NO3)+2 Cl- 0.191
Sm(NO3)+2 ClO4- 0.39
Sm(OH)+2 Cl- -0.04
Sm(OH)+2 ClO4- 0.39
Sm(OH)2+ Cl- -0.29
Sm(OH)2+ ClO4- 0.17
Sm(SO4)+ Cl- 0.157
Sm(SO4)+ ClO4- 0.22
Sm(SO4)2- Na+ -0.05
Sm+3 Cl- 0.23
Sm+3 ClO4- 0.49
SmCl+2 Cl- 0.191
SmCl+2 ClO4- 0.39
SmF+2 Cl- 0.191
SmF+2 ClO4- 0.39
Sn(OH)+ ClO4- -0.07
Sn(OH)3- Na+ 0.22
Sn+2 Cl- 0.14
Sn+2 ClO4- 0.19
Sn+4 ClO4- 0.7
Sn3(OH)4+2 ClO4- -0.02
SnBr+ ClO4- 0.17
SnBr3- Na+ 0.16
SnCl+ ClO4- 0.08
SnCl3- Na+ 0.04
SnF+ ClO4- 0.14
Th(CO3)5-6 Na+ -0.3
Th(H2PO4)+3 ClO4- 0.5
Th(H2PO4)2+2 ClO4- 0.4
Th(H3PO4)(H2PO4)+3 ClO4- 0.5
Th(H3PO4)+4 ClO4- 0.7
Th(NO3)+3 ClO4- 0.56
Th(NO3)+3 NO3- 0.56
Th(NO3)2+2 ClO4- 0.43
Th(NO3)2+2 NO3- 0.43
Th(OH)(CO3)4-5 Na+ -0.22
Th(OH)+3 Cl- 0.19
Th(OH)+3 ClO4- 0.48
Th(OH)+3 NO3- 0.2
Th(OH)2(CO3)2-2 Na+ -0.1
Th(OH)2+2 Cl- 0.13
Th(OH)2+2 ClO4- 0.33
Th(OH)2+2 NO3- 0.1
Th(OH)3(CO3)- Na+ -0.05
Th(OH)3+ Cl- 0.06
Th(OH)3+ ClO4- 0.15
Th(OH)3+ NO3- 0.05
Th(OH)4(CO3)-2 Na+ -0.1
Th(SO4)+2 Cl- 0.14
Th(SO4)+2 ClO4- 0.3
Th(SO4)3-2 Na+ -0.091
Th+4 Cl- 0.7

Th+4 ClO4- 0.7
Th+4 NO3- 0.7
Th2(OH)2+6 Cl- 0.4
Th2(OH)2+6 ClO4- 1.22
Th2(OH)2+6 NO3- 0.69
Th2(OH)3+5 Cl- 0.29
Th2(OH)3+5 ClO4- 0.91
Th2(OH)3+5 NO3- 0.69
Th4(OH)12+4 Cl- 0.25
Th4(OH)12+4 ClO4- 0.56
Th4(OH)12+4 NO3- 0.42
Th4(OH)8+8 Cl- 0.7
Th4(OH)8+8 ClO4- 1.69
Th4(OH)8+8 NO3- 1.59
Th6(OH)14+10 Cl- 0.83
Th6(OH)14+10 ClO4- 2.2
Th6(OH)14+10 NO3- 2.9
Th6(OH)15+9 Cl- 0.72
Th6(OH)15+9 ClO4- 1.85
Th6(OH)15+9 NO3- 2.2
ThCl+3 Cl- 0.62
ThCl+3 ClO4- 0.62
ThF+3 ClO4- 0.48
ThF+3 NO3- 0.25
ThF2+2 ClO4- 0.3
ThF2+2 NO3- 0.15
ThF3+ ClO4- 0.1
ThF3+ NO3- 0
U(CO3)4-4 Na+ -0.09
U(CO3)5-6 Na+ -0.3
U(NO3)+3 ClO4- 0.62
U(NO3)2+2 Cl- 0.236
U(NO3)2+2 ClO4- 0.49
U(OH)+3 Cl- 0.2
U(OH)+3 ClO4- 0.48
U(OH)2+2 Cl- 0.1
U(OH)3+ Cl- 0.05
U(SO4)+2 Cl- 0.149
U(SO4)+2 ClO4- 0.3
U+3 Cl- 0.23
U+3 ClO4- 0.49
U+4 Cl- 0.36
U+4 ClO4- 0.76
UBr+3 ClO4- 0.52
UCl+3 Cl- 0.1
UCl+3 ClO4- 0.5
UF+3 Cl- 0.1
UF+3 ClO4- 0.48
UF2+2 Cl- 0.149
UF2+2 ClO4- 0.3
UF3+ Cl- 0.1
UF3+ ClO4- 0.1
UI+3 ClO4- 0.55
UI+3 NO3- 0.56
UO2(CO3)2-2 Na+ -0.02
UO2(CO3)3-4 Na+ -0.01
UO2(CO3)3-5 Na+ -0.62
UO2(Cit)- Na+ -0.11
UO2(NO3)+ Cl- 0.22
UO2(NO3)+ ClO4- 0.33
UO2(OH)+ Cl- -0.003
UO2(OH)+ ClO4- -0.06
UO2(OH)+ NO3- 0.51
UO2(OH)3- Na+ -0.09
UO2(Ox)2-2 Na+ -0.18
UO2(Ox)3-4 Na+ -0.01
UO2(SO4)2-2 Na+ -0.12
UO2+ Cl- 0.18
UO2+ ClO4- 0.26
UO2+2 Cl- 0.46
UO2+2 ClO4- 0.46
UO2+2 NO3- 0.46
UO2Br+ Cl- 0.169
UO2Br+ ClO4- 0.24
UO2Cl+ Cl- 0.22
UO2Cl+ ClO4- 0.33
UO2F+ Cl- 0.04
UO2F+ ClO4- 0.28
UO2F3- Na+ -0.14
UO2F4-2 Na+ -0.3
Zn+2 Cl- 0.163
Zn+2 ClO4- 0.33
Zn+2 NO3- 0.16
Zr(CO3)4-4 Na+ -0.09
Zr(NO3)2+2 ClO4- 0.84
Zr(OH)+3 ClO4- 0.57
Zr(OH)2+2 ClO4- 0.62
Zr(OH)6-2 Na+ -0.1
Zr+4 Cl- 0.89
Zr+4 ClO4- 0.89
Zr+4 NO3- 0.89
Zr4(OH)15+ ClO4- 0.09
ZrCl+3 ClO4- 0.87
ZrCl2+2 ClO4- 0.84
ZrF+3 ClO4- 0.63
ZrF2+2 ClO4- 0.47
ZrF5- Na+ -0.14
ZrF6-2 Na+ -0.15
ZrNO3+3 ClO4- 0.88

```
SOLUTION_SPECIES

1.000H2O     = H2O
    log_k     0.000
    delta_h   0.000     #kJ/mol
    # Enthalpy of formation:                  #kJ/mol
    -analytic 0E+0 0E+0 0E+0 0E+0 0E+0

1.000H+     = H+
    log_k     0.000
    delta_h   0.000     #kJ/mol
    # Enthalpy of formation:          0       #kJ/mol       #89COX/WAG
    -analytic 0E+0 0E+0 0E+0 0E+0 0E+0

1.000e-     = e-
    log_k     0.000
    delta_h   0.000     #kJ/mol
    # Enthalpy of formation:          0       #kJ/mol       #89COX/WAG
    -analytic 0E+0 0E+0 0E+0 0E+0 0E+0

1.000Nb(OH)6-     = Nb(OH)6-
    log_k     0.000
    delta_h   0.000     #kJ/mol
    # Enthalpy of formation:          -1925.665       #kJ/mol
    -analytic 0E+0 0E+0 0E+0 0E+0 0E+0

1.000Ox-2     = Ox-2
    log_k     0.000
    delta_h   0.000     #kJ/mol
    # Enthalpy of formation:          -830.66       #kJ/mol
    -analytic 0E+0 0E+0 0E+0 0E+0 0E+0

1.000H2O     - 0.500O2     = H2
    log_k   -46.070
    delta_h  275.563    #kJ/mol
    # Enthalpy of formation:          -4.2       #kJ/mol       #82WAG/EVA
    -analytic 2.20649E+0 0E+0 -1.43936E+4 0E+0 0E+0

- 4.000H+     - 4.000e-     + 2.000H2O     = O2
    log_k   -85.980     #89COX/WAG
    delta_h  559.526    #kJ/mol
    # Enthalpy of formation:          -12.134       #kJ/mol       #01SCH/SHO
    -analytic 1.20446E+1 0E+0 -2.9226E+4 0E+0 0E+0
3.000H+     + 1.000Nb(OH)6-     - 3.000H2O     = Nb(OH)3+2
    log_k     7.500     #97PEI/NGU
    delta_h  -10.230    #kJ/mol       #97PEI/NGU
    # Enthalpy of formation:          -1078.405       #kJ/mol
    -analytic 5.70778E+0 0E+0 5.34349E+2 0E+0 0E+0

2.000H+     + 1.000Nb(OH)6-     - 2.000H2O     = Nb(OH)4+
    log_k     6.640     #97PEI/NGU
    delta_h  -35.350    #kJ/mol       #97PEI/NGU
    # Enthalpy of formation:          -1389.355       #kJ/mol
    -analytic 4.46955E-1 0E+0 1.84646E+3 0E+0 0E+0

1.000H+     + 1.000Nb(OH)6-     - 1.000H2O     = Nb(OH)5
    log_k     5.080     #97PEI/NGU
    delta_h  -13.390    #kJ/mol       #97PEI/NGU
    # Enthalpy of formation:          -1653.225       #kJ/mol
    -analytic 2.73418E+0 0E+0 6.99407E+2 0E+0 0E+0
```

```
- 1.000H+     + 1.000Nb(OH)6-     + 1.000H2O     = Nb(OH)7-2
    log_k    -8.880     #97PEI/NGU
    delta_h  10.170     #kJ/mol        #97PEI/NGU
    # Enthalpy of formation:           -2201.325        #kJ/mol
    -analytic -7.0983E+0 0E+0 -5.31215E+2 0E+0 0E+0

5.000H+     + 1.000Nb(OH)6-     + 1.000Cit-3     - 4.000H2O     = NbO2(H3Cit)+
    log_k    25.640     #95AKR/BOU
    # delta_h  0.000     #kJ/mol
    # Enthalpy of formation:                   #kJ/mol
    -analytic 2.564E+1 0E+0 0E+0 0E+0 0E+0

3.000H+     + 1.000Nb(OH)6-     + 1.000Ox-2     - 4.000H2O     = NbO2(HOx)
    log_k    13.700     #95AKR/BOU
    # delta_h  0.000     #kJ/mol
    # Enthalpy of formation:                   #kJ/mol
    -analytic 1.37E+1 0E+0 0E+0 0E+0 0E+0

4.000H+     + 1.000Nb(OH)6-     + 2.000Ox-2     - 4.000H2O     = NbO2(HOx)2-
    log_k    20.960     #95AKR/BOU
    # delta_h  0.000     #kJ/mol
    # Enthalpy of formation:                   #kJ/mol
    -analytic 2.096E+1 0E+0 0E+0 0E+0 0E+0

2.000H+     + 1.000Nb(OH)6-     + 1.000Ox-2     - 4.000H2O     = NbO2(Ox)-
    log_k    10.940     #95AKR/BOU
    # delta_h  0.000     #kJ/mol
    # Enthalpy of formation:                   #kJ/mol
    -analytic 1.094E+1 0E+0 0E+0 0E+0 0E+0


PHASES

Nb(cr)
Nb     = 1.000H+     + 1.000Nb(OH)6-     - 3.500H2O     - 1.250O2
    log_k   149.295
    delta_h -910.093    #kJ/mol
    # Enthalpy of formation:           0        #kJ/mol        #82WAG/EVA
    -analytic -1.01461E+1 0E+0 4.75374E+4 0E+0 0E+0

Nb2O5(s)
Nb2O5     = 2.000H+     + 2.000Nb(OH)6-     - 7.000H2O
    log_k   -28.380     #97PEI/NGU
    delta_h  52.400     #kJ/mol        #97PEI/NGU
    # Enthalpy of formation:           -1902.92       #kJ/mol
    -analytic -1.91999E+1 0E+0 -2.73704E+3 0E+0 0E+0


# PMATCH GASES

H2(g)
H2     = 1.000H2O     - 0.500O2
    log_k    42.990
    delta_h -279.763    #kJ/mol
    # Enthalpy of formation:           0        #kJ/mol        #89COX/WAG
    -analytic -6.0223E+0 0E+0 1.4613E+4 0E+0 0E+0

H2O(g)
H2O     = 1.000H2O
    log_k     1.500
    delta_h  -44.004    #kJ/mol
```

```
    # Enthalpy of formation:           -241.826        #kJ/mol        #89COX/WAG
    -analytic -6.20916E+0 0E+0 2.29849E+3 0E+0 0E+0

O2(g)
O2     = 1.000O2
    log_k    -2.900
    delta_h  -12.134    #kJ/mol
    # Enthalpy of formation:           0        #kJ/mol
    -analytic -5.02578E+0 0E+0 6.33802E+2 0E+0 0E+0
```
